# Exploring the Natural Origins of SARS-CoV-2 in the Light of Recombination

**DOI:** 10.1093/gbe/evac018

**Published:** 2022-02-08

**Authors:** Spyros Lytras, Joseph Hughes, Darren Martin, Phillip Swanepoel, Arné de Klerk, Rentia Lourens, Sergei L Kosakovsky Pond, Wei Xia, Xiaowei Jiang, David L Robertson

**Affiliations:** 1 MRC-University of Glasgow Centre for Virus Research, Glasgow, United Kingdom; 2 Computational Biology Division, Department of Integrative Biomedical Sciences, University of Cape Town, South Africa; 3 Division of Neurosurgery, Department of Surgery, Neuroscience Institute, University of Cape Town, South Africa; 4 Department of Biology, Institute for Genomics and Evolutionary Medicine, Temple University, USA; 5 National School of Agricultural Institution and Development, South China Agricultural University, Guangzhou, China; 6 Department of Biological Sciences, Xi’an Jiaotong-Liverpool University (XJTLU), Suzhou, China

**Keywords:** SARS-CoV-2, *Sarbecoviruses*, bats, origin, COVID-19, host range, coronaviruses, recombination, *Rhinolophus*, pangolins

## Abstract

The lack of an identifiable intermediate host species for the proximal animal ancestor of SARS-CoV-2, and the large geographical distance between Wuhan and where the closest evolutionary related coronaviruses circulating in horseshoe bats (members of the *Sarbecovirus* subgenus) have been identified, is fueling speculation on the natural origins of SARS-CoV-2. We performed a comprehensive phylogenetic study on SARS-CoV-2 and all the related bat and pangolin sarbecoviruses sampled so far. Determining the likely recombination events reveals a highly reticulate evolutionary history within this group of coronaviruses. Distribution of the inferred recombination events is nonrandom with evidence that Spike, the main target for humoral immunity, is beside a recombination hotspot likely driving antigenic shift events in the ancestry of bat sarbecoviruses. Coupled with the geographic ranges of their hosts and the sampling locations, across southern China, and into Southeast Asia, we confirm that horseshoe bats, *Rhinolophus*, are the likely reservoir species for the SARS-CoV-2 progenitor. By tracing the recombinant sequence patterns, we conclude that there has been relatively recent geographic movement and cocirculation of these viruses’ ancestors, extending across their bat host ranges in China and Southeast Asia over the last 100 years. We confirm that a direct proximal ancestor to SARS-CoV-2 has not yet been sampled, since the closest known relatives collected in Yunnan shared a common ancestor with SARS-CoV-2 approximately 40 years ago. Our analysis highlights the need for dramatically more wildlife sampling to: 1) pinpoint the exact origins of SARS-CoV-2’s animal progenitor, 2) the intermediate species that facilitated transmission from bats to humans (if there is one), and 3) survey the extent of the diversity in the related sarbecoviruses’ phylogeny that present high risk for future spillovers.

Significance

The origin of SARS-CoV-2 can be unambiguously traced to horseshoe bats, genus *Rhinolophus*. SARS-related coronaviruses, like SARS-CoV-2, are dispersed over a large geographical area across southern China and Southeast Asia. They have undergone extensive recombination throughout their evolutionary history indicating frequent transmission among their *Rhinolophus* host species. Breakpoint patterns are consistent with recombination hotspots in the coronavirus genome, particularly upstream of the spike open reading frame with a coldspot in S1. Accounting for these recombination patterns is important when inferring relatedness to SARS-CoV-2.

## Introduction

Two years since the emergence of SARS-CoV-2, the origins of this new pandemic human coronavirus remain uncertain. First detected in association with an unusual respiratory disease outbreak in December 2019 in Wuhan city, Hubei province, China (Li, Guan, et al. 2020) no definitive progenitor of animal origin has been identified. The first reports of the initial outbreak were linked to the Huanan animal and seafood market (WHO 2021; Worobey 2021) and, while there are some cases with no identifiable association to this location, this is not so surprising given that so many cases are either mild or asymptomatic (Lytras et al. 2021), and it is possible multiple spillover events at animal markets in Wuhan were involved (Holmes et al. 2021; WHO 2021). Since the 2020 coronavirus pandemic began, both metagenomic and focused sequencing efforts have uncovered a number of viruses related to SARS-CoV-2, retrieved from locations in China and Southeast Asia (Hu et al. 2018; Zhou, Chen, et al. 2020; Zhou, Yang, et al. 2020; Delaune et al. 2021; Li et al. 2021; Wacharapluesadee et al. 2021; Zhou et al. 2021). Several of these sarbecoviruses are recombinants necessitating careful analysis as the presence of mosaic genomes violates the assumption of a single evolutionary history, key to reliable phylogenetic inference from mutation patterns in molecular data.

**Fig. 1:**
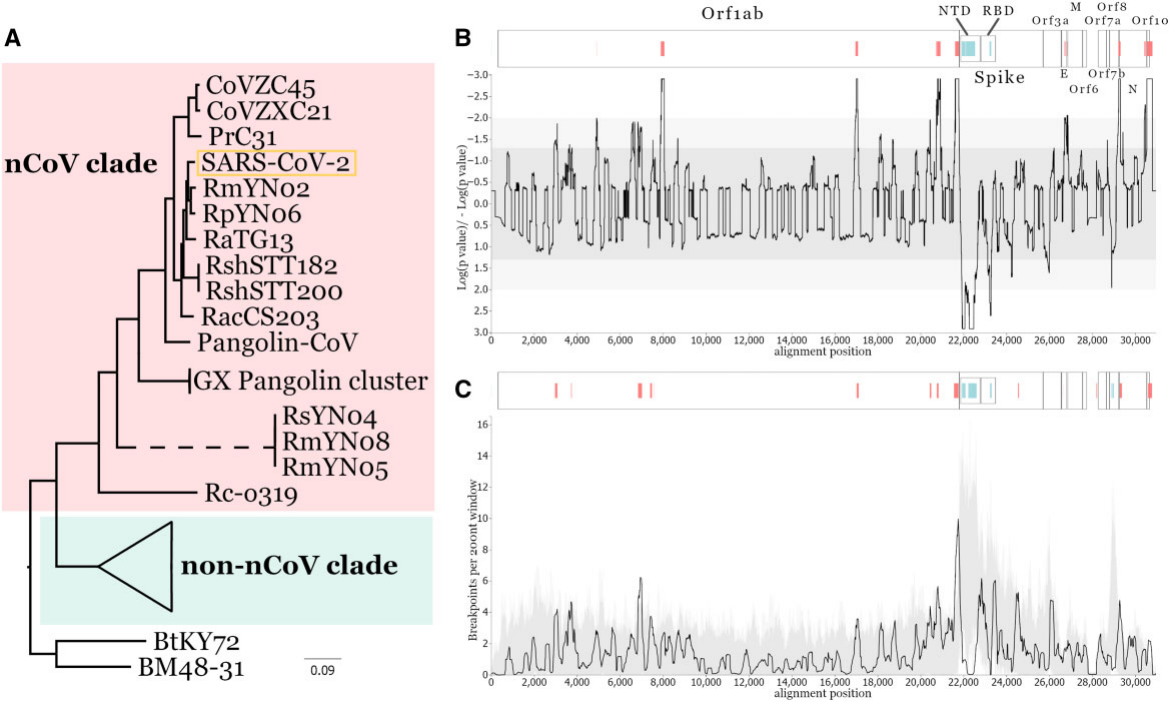
Recombination-minimized phylogeny and recombination hot-/coldspots. Maximum likelihood phylogeny inferred from a recombination-free whole-genome alignment of the 78 *Sarbecoviruses* (*A*), see Materials and Methods. The non-nCoV/SARS-CoV clade is collapsed for clarity. All nodes presented have bootstrap confidence values above 90%. Distribution of recombination hot- and coldspots across the alignment based on the RRT (*B*) and the BDT (*C*) methods. For both plots, light and dark gray represent 95% and 99% confidence intervals of expected recombination breakpoint clustering under random recombination. Peaks above the shaded area represent recombination hotspots and drops below represent coldspots, annotated on the corresponding ORF genome schematic above each plot by vertical red and blue lines, respectively. All ORF names and the NTD and RBD encoding regions of Spike are also annotated on the schematics.

SARS-CoV-2, responsible for COVID-19, and SARS-CoV, the causative agent of the SARS outbreak in 2002–2003, are both members of the species *Severe acute respiratory syndrome-related coronavirus* (SARSr-CoV) that forms the sole member of the *Sarbecovirus* subgenus of *Betacoronaviruses* (Gorbalenya et al. 2020)—a group of viruses which have been primarily found in horseshoe bats (family *Rhinolophidae*). Coronaviruses are known to recombine with one another during mixed infections (Graham and Baric 2010; Boni et al. 2020). Here, we comprehensively characterize the recombinant nature of the SARS-CoV-2-like coronaviruses sampled so far, focusing specifically on the phylogenetic clade of sarbecoviruses that SARS-CoV-2 is a member of; hereafter referred to as the “nCoV” clade (fig. 1*A*) (MacLean et al. 2021). To maintain the focus on this clade from which SARS-CoV-2 emerged, we broadly refer to all other *Sarbecovirus* subclades as ‘non-nCoV’. We present evidence of recombination and several hotspot locations where inferred recombination breakpoints are overrepresented. By comparing the phylogenies inferred for putatively nonrecombinant regions of the genome (i.e., best estimates of SARS-CoV-2 and related sarbecoviruses true evolutionary history) with the viruses’ sampling locations and their host’s geographic range locations, we provide a detailed understanding of the recent evolutionary histories of SARS-CoV-2’s closest known relatives including relative divergence times.

## Results and Discussion

### Hotspots of Recombination

For a whole-genome alignment of the set of known complete genomes from 78 members of the *Sarbecovirus* subgenus (including a single representative of SARS-CoV and SARS-CoV-2; supplementary table S1, Supplementary Material online), we performed an initial recombination breakpoint analysis with RDP5 (see Materials and Methods) and identified 160 unique recombination events in all the bat and pangolin-derived virus genomes. To infer a reliable phylogeny of the sarbecoviruses, we removed all regions with evidence for a recombination history from the genome alignment. This reconstructed nonrecombinant phylogeny (fig. 1*A*) includes a total of 19 nonhuman viruses that comprise the nCoV clade that SARS-CoV-2 is a member of, a sister lineage to the non-nCoV clade SARS-CoV is part of, first emerged from in 2002.

Using the set of breakpoints inferred by RDP5, we tested for significant clustering of recombination events at specific regions of the genome, suggestive of recombination hot- or coldspots. Two permutation-based recombination breakpoint clustering tests were performed: 1) a “breakpoint distribution test” (BDT) that explicitly accounts for the underlying uncertainties in the positions of identified breakpoint positions (Heath et al. 2006) and 2) a “recombinant region test” (RRT) that focuses on point estimates of recombination breakpoint pairs that define recombination events and explicitly accounts for region-to-region variations in the detectability of recombination events (Simon-Loriere et al. 2009). Both tests provided support for the presence of several recombination hotspots: seven in the BDT and nine in the RRT analysis, assuming close locations are giving rise to the same peak (fig. 1*B* and *C*), and recombination refractory regions in the NTD and RBD domains of the Spike gene and within open-reading frame (ORF)8 (fig. 1*C*).

It is possible that all genomic regions where these breakpoint clusters are detected have elevated recombination rates, linked to the molecular mechanisms likely responsible for recombination (Sola et al. 2015). However, simulations of recombination patterns—in genomes with similar degrees of diversity and numbers of detectable recombination events to the genomes analyzed here—indicate that within such a data set we might expect to find, on average, two to three such clusters even in the absence of any recombination hotspots (see Materials and Methods; supplementary table S2, Supplementary Material online). Therefore, none of the identified breakpoint clusters can be definitively attributed to underlying variations in recombination rates at the genome sites where the clusters are identified. Nonetheless, the distribution of recombination breakpoints is clearly nonuniform across the *Sarbecovirus* genomes, and this nonuniformity is consistent with the presence of recombination hotspots. To independently validate the results of this analysis, we also performed a simple permutation test for clustering in the recombination breakpoints inferred by the Genetic Algorithm for Recombination Detection (GARD) analysis (see below, supplementary fig. S2, Supplementary Material online). Even though this test would not identify potential hotspots in proximal genomic locations (due to the nature of the GARD method which is expected to identify focused recombination hotspots as a single recombination breakpoint), it confirms the recombination hotspots within the Spike ORF (alignment positions 24174–24648, supplementary fig. S2, Supplementary Material online—consistent with the BDT results, fig. 1*C*) and at the start of the N ORF (alignment positions 29388–29862, supplementary fig. S2, Supplementary Material online, consistent with both RRT and BDT results, fig. 1*C*).

Interestingly the pattern of potential hotspots near the Spike ORF has also been noted in previous research (Bobay et al. 2020). Although selective pressure underlying recombinant regions cannot be assessed in this analysis, antigenic selection—for immune escape—and/or selection associated with switches in host receptor specificity and efficiency—that is, antigenic shift—are two probable candidate drivers of the observed recombination patterns, consistent with the known immunodominance of the Spike NTD and RBD regions (Walls et al. 2020). It is clearly important to account for these complex recombination patterns when examining the evolutionary history of these pathogens, since multiple evolutionary histories can be inferred from the single whole-genome alignment. As SARS-CoV-2 continues circulating in humans and mutations increase its sequence diversity, identifying SARS-CoV-2 recombination events will become easier and increasingly more important to monitor (Jackson et al. 2021).

### Recombination Patterns between SARS-CoV-2 Relatives

To reconstruct a reliable phylogeny for a set of viruses, sufficient information needs to be present in the underlying sequence alignment. Thus, even though a whole-genome alignment can be split into shorter subalignments with the aim of getting rid of all independent recombination events, it is unlikely that all subalignments can produce reliable phylogenies. To overcome this trade-off, we performed a secondary, more conservative, recombination analysis using GARD and identified the locations of 21 recombination breakpoints that strongly impact the inferred phylogenetic relationships of the analyzed sequences when mosaic patterns are ignored (supplementary table S3, Supplementary Material online). In contrast to the RDP5 method used above for assessing breakpoint clustering, the GARD method focuses on extracting recombination signal for the entire alignment, and so is better suited for producing putatively nonrecombinant phylogenies. We then determined the phylogenetic relationships of the viral sequences in each of the 22 putatively nonrecombinant genome regions bounded by each identifiable breakpoint (fig. 3*A*). The 20 nCoV viruses identified in the nonrecombinant whole-genome phylogeny above (fig. 1*A*) were used to inform the clade annotation for the 22 new nonrecombinant phylogenies.

**Fig. 2:**
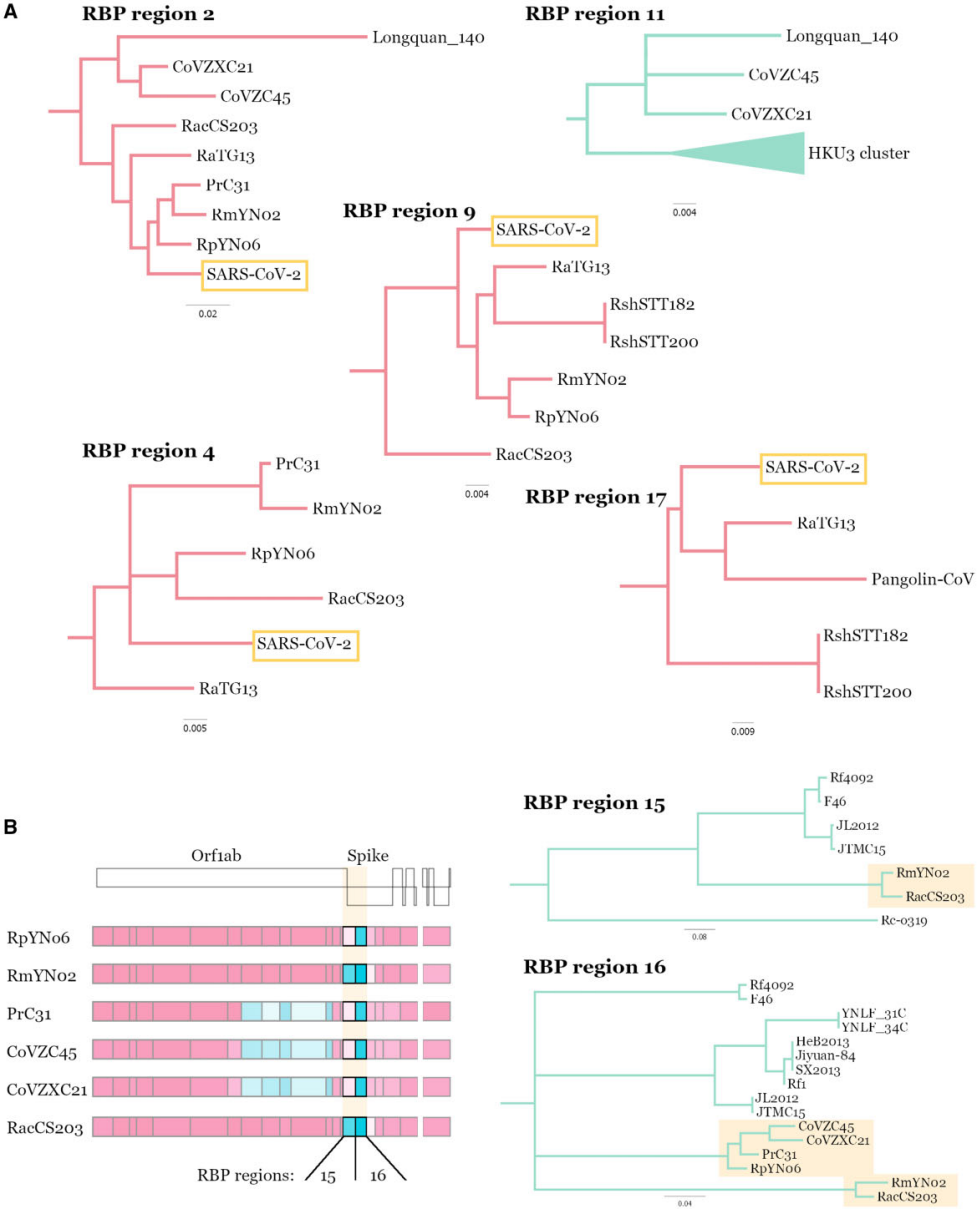
Nonrecombinant topologies of SARS-CoV-2 relatives. Zoomed in regions of selected RBP region maximum likelihood phylogenies (*A*). Branches within the nCoV clade are colored in red and outside the nCoV clade in green. Genome schematics of close SARS-CoV-2 relatives with recombinant Spike regions (*B*). RBP regions 15 and 16 are highlighted and the non-nCoV subclades of the maximum likelihood phylogenies containing the relevant viruses are presented. The coloring of nonrecombinant segments indicates patristic distance to SARS-CoV-2 (see fig. 3 legend). Nodes with bootstrap confidence values below 80% have been collapsed.

The two genetically closest relatives of SARS-CoV-2 that were identified shortly after its emergence were the bat sarbecoviruses, RaTG13 and subsequently RmYN02, both from samples collected in Yunnan (Zhou, Chen, et al. 2020; Zhou, Yang, et al. 2020). We find RmYN02 shares a common ancestor with SARS-CoV-2 about 40 years ago and RaTG13—about 50 years ago (fig. 4*A*) consistent with previous estimates (Boni et al. 2020; MacLean et al. 2021; Wang et al. 2021). Although SARS-CoV-2 is most similar to RmYN02 across most of its genome, the region corresponding to the first half of the RmYN02 Spike ORF appears to have been derived through recombination from a parental sequence residing outside the nCoV clade (fig. 1*A*). Two more viruses very recently identified in Yunnan, RpYN06 and PrC31, are most closely related to RmYN02 for part of their genomes (Li et al. 2021; Zhou et al. 2021). In the portion of the genome corresponding to recombination breakpoint partitioned (RBP) regions 2–5, the three Yunnan viruses (RmYN02, RpYN06, and PrC31) cluster with strong support in a sister clade to SARS-CoV-2 (fig. 2*A* and supplementary fig. S1, Supplementary Material online). This pattern suggests that bat sampling efforts in Yunnan have uncovered a related viral population that has relatively recently shared a common ancestor with SARS-CoV-2’s proximal ancestor. Molecular dating of the RBP region 5 phylogeny (corresponding to the C-terminal part of nsp3; fig. 4*A*) indicates that this “Yunnan cluster” shared a common ancestor with SARS-CoV-2 around 1982 (95% HPD: 1970–1994). This analysis further allows us to date the node between PrC31 and RmYN02 to 2005 (95% HPD: 1998–2010), which is one of the most recent nodes in the phylogeny (fig. 4*A*).

**Fig. 3:**
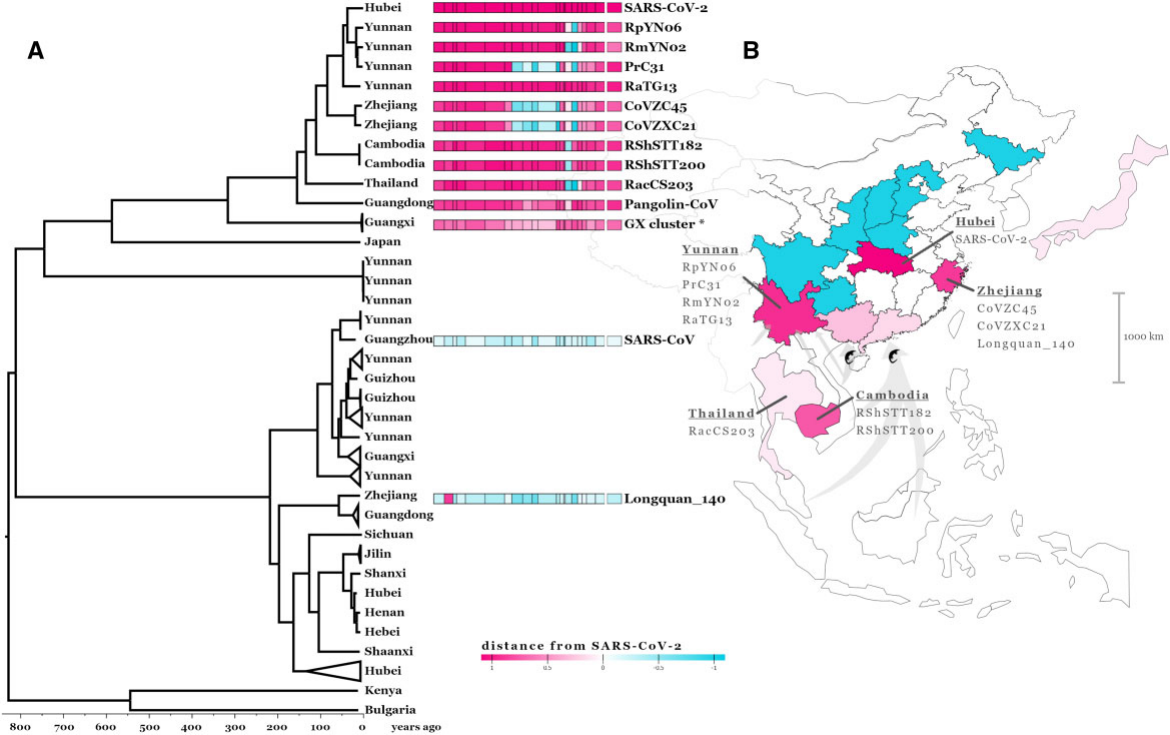
Recombination analysis and geographic distribution of *Sarbecoviruses*. Maximum clade credibility (MCC) dated phylogeny of RBP region 5 of 78 *Sarbecoviruses* (*A*). All tips are annotated with the geographic region the viruses have been sampled in and notable viruses are annotated with genome schematics separated into the 22 inferred RBP regions, each colored based on phylogenetic distance from SARS-CoV-2 (see scale and Materials and Methods). RBP region 21 has been removed from the schematic due to limited phylogenetic information in the alignment. The GX cluster annotated with an asterisk contains the five pangolin coronaviruses collected in Guangxi. Map of East Asia with geographic regions (provinces within China, countries outside China) colored based on *Sarbecoviruses* sampling (*B*): blue for regions with only non-nCoV clade samples, pink for regions where nCoV viruses have been sampled. Shading in the nCoV regions corresponds to phylogenetic distance from SARS-CoV-2 (see scale). Notable nCoV viruses and pangolin trafficking routes (adapted from Xu et al. [2016]) are annotated onto the map.

**Fig. 4:**
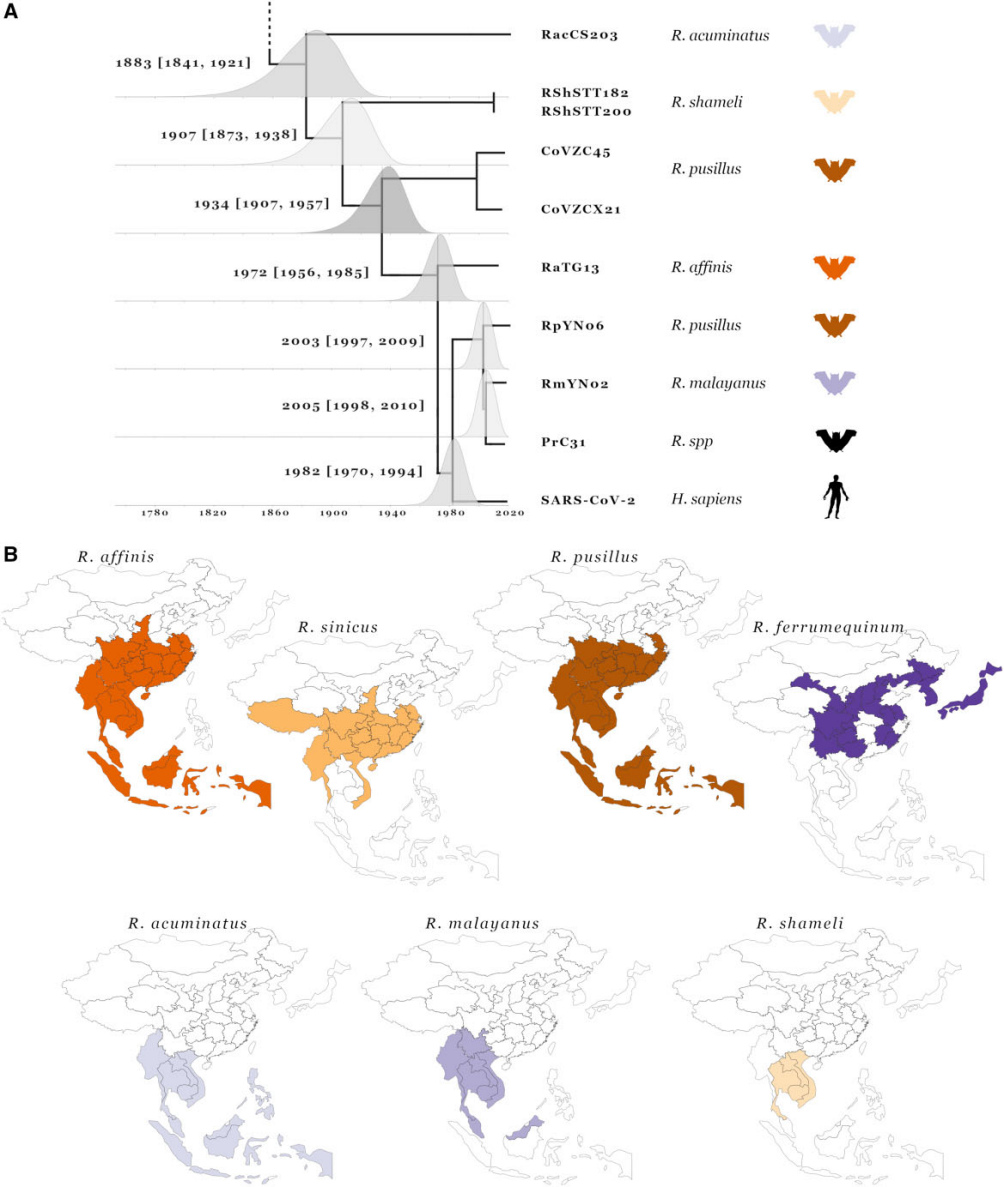
Molecular dating and *Rhinolophus* host geographic distributions. Tip-dated Bayesian phylogeny of RBP region 5 showing the nine closest relatives to SARS-CoV-2 (*A*). Tree nodes have been adjusted to the mean age estimates and posterior distributions are shown for each node with mean age estimate and 95% HPD confidence intervals presented to their left. Tips are annotated with the host species they were sampled in, bat silhouette colors correspond to panel (*B*). Geographic ranges of *Rhinolophus* species the SARS-CoV-2 closest relatives have been sampled in (*B*). Maps are restricted to East Asia and separated into province-level within China and country-level outside China.

The recombination analysis, however, reveals a much more complex evolutionary history for the rest of the PrC31 genome (Li et al. 2021). As seen in the consensus whole-genome phylogeny (fig. 1*A*), most of its genome clusters with viruses CoVZC45 and CoVZXC21 sampled in Zhejiang, a coastal province in East China (Lin et al. 2017; Hu et al. 2018). Across the majority of their genomes (excluding segments of Orf1ab and Spike) these viruses are members of the nCoV clade and share a common ancestor with SARS-CoV-2 that existed before 1934 (95% HPD: 1907–1957) according to molecular dating of RBP region 5 (fig. 4*A*). However, in RBP regions 8–12, the sequences of these viruses cluster outside the nCoV clade, and are most closely related to Zhejiang virus Longquan_140 and the HKU3 set of closely related bat sarbecoviruses sampled in Hong Kong (bordering Guangdong province) (fig. 2*A* and supplementary fig. S1, Supplementary Material online). The link between SARS-CoV-2’s closest relatives and viral populations in the southeast of South China becomes even more apparent in the phylogeny of RBP region 2 where Longquan_140 clusters within the nCoV clade along with CoVZC45 and CoVZXC21 (fig. 2*A* and supplementary fig. S1, Supplementary Material online, RBP region 2 tree). These relationships indicate ancestral movement of the nCoV viruses across large geographic ranges in China, spanning Yunnan in southwest China and Zhejiang on the east coast (fig. 3*B*).

As more countries initiate wildlife-infecting coronavirus sampling and sequencing efforts, the geographic range of the nCoV clade linked to bat host species will be further refined, evident from the recent reporting of bat sarbecoviruses closely related to SARS-CoV-2 from: 1) two samples collected in Cambodia from *Rhinolophusshameli* (RShSTT182 and RShSTT200) confirmed by whole-genome analysis (Delaune et al. 2021), and 2) five bat samples from *Rhinolophusacuminatus* collected in Thailand with one fully sequenced genome of virus RacCS203 (Wacharapluesadee et al. 2021). These viruses are, after the China sampled CoVs mentioned above, the next closest relatives to SARS-CoV-2 with common ancestor age estimates (using RBP region 5) around 1907 (95% HPD: 1873–1938) and 1883 (95% HPD: 1841–1921), respectively (fig. 4*A*). Similar to the other nCoV viruses, the recombination analysis uncovers more intricate phylogenetic relations for some parts of the genome. Notably, RShSTT182 and RShSTT200, despite being sampled in Cambodia, cluster with RaTG13 for RBP regions 8 and 9 (fig. 2*A* and supplementary fig. S1, Supplementary Material online), while in RBP region 4 of the genome RacCS203, from Thailand, clusters together with SARS-CoV-2 within the Yunnan clade (fig. 2*A*). This indicates that cocirculation and recombination between these viruses in the last few centuries is responsible for the observed patterns in their inferred evolutionary history, despite the current geographic range of at least 2,500km. This wide distribution of related viruses, including shared recombination breakpoints, highlights an important feature of bat species: Their frequently overlapping/sympatric ranges will provide ample opportunities for transmissions of viral variants from one bat species (or subspecies) to another.

Consistent with the Spike S1 recombination hotspots revealed in the initial analysis (fig. 1*B* and *C*), closest relatives of SARS-CoV-2 presented here have non-nCoV derived recombinant sequences at the start of the Spike gene (fig. 2*B*). Despite one collected from Yunnan, China and the other from Thailand, viruses RmYN02 and RacCS203 share a closely related non-nCoV sequence in RBP regions 15 and 16 (encompassing the Spike NTD and RBD, respectively; fig. 2*B*) having a distinct RBD compared with that of SARS-CoV-2. On the other hand, viruses RpYN06, PrC31, CoVZC45, and CoVZXC21 cluster within the nCoV clade for region 15 but, similar to the RmYN02 and RacCS203, form a distinct cluster in the non-nCoV clade for region 16 (fig. 2*B*; Wells et al. 2021). We speculate that some of the apparent patterns of recombination-mediated exchange between nCoV and non-nCoV viruses can be partly explained by sequential recombination, that is, “overprinting” of recombination events involving different ancestral parental viruses. This will occur when an nCoV virus acquires a non-nCoV genomic sequence through ancestral recombination but its progenitors cocirculating with other nCoV viruses incur subsequent recombination events that overlap portions of the original non-nCoV recombinant sequence, producing the more complex “patchy” patterns we see in the currently sampled viruses. Note, overprinting of recombination regions will result in reduced confidence in the breakpoints at deeper nodes in the phylogeny.

The finding that Sunda (also known as Malayan) pangolins, *Manis javanica*, nonnative to China, are the other mammal species from which nCoV sarbecoviruses have been sampled in Guangxi and Guangdong provinces in South China (Lam et al. 2020; Xiao et al. 2020), indicates these animals are likely being infected in this part of the country (fig. 3*B*). Pangolins are one of the most frequently trafficked animals with multiple smuggling routes leading to southern China (Xu et al. 2016). The most common routes involve moving the animals from Southeast Asia (Myanmar, Malaysia, Laos, Indonesia, Vietnam) to Guangxi, Guangdong, and Yunnan. The most likely scenario that is consistent with both the reported respiratory distress that the sampled pangolins exhibited (Liu et al. 2019; Xiao et al. 2020) and the lack of confirmed CoV infections among Sunda pangolins in Malaysia (Lee et al. 2020), is that the viruses obtained from these animals infected them (presumably from bat sources) after they were trafficked into southern China. Still, serological data of trafficked Sunda pangolins could suggest potential circulation of sarbecoviruses in the animals’ wild populations (Wacharapluesadee et al. 2021).

Although the recombination patterns inferred in the pangolin-derived virus genomes seem to be less complex than those of the bat nCoV genomes, the Guangdong Pangolin-CoV has a Spike receptor binding domain that is most similar to that of SARS-CoV-2. This finding was highlighted by Li, Giorgi, et al. (2020) and attributed to recombination between the SARS-CoV-2 and Pangolin-CoV proximal ancestors. However, based on the nucleotide divergence between the two viruses in this short Spike segment, the most likely explanation is recombination in an ancestor of RaTG13, making it more divergent than Pangolin-CoV compared with SARS-CoV-2 (Boni et al. 2020) (reflected in region 17, fig. 2*A*). The susceptibility of pangolins to an apparently new human coronavirus is not surprising given the well-documented generalist nature of SARS-CoV-2 (Conceicao et al. 2020), which has been found to readily transmit to multiple mammals with similar ACE2 receptors, most notably, on mink farms (Oude Munnink et al. 2021).

### Overlapping Horseshoe Bat Ranges

Considering that almost all sarbecoviruses have been sampled in related horseshoe bat host species, with ranges that span different regions where nCoV clade viruses have been collected (fig. 4*B*), these bat populations should be prioritized for sampling. For example, the least horseshoe bat species, *Rhinolophuspusillus*, is sufficiently dispersed across China to account for the geographical spread of: 1) bat sarbecovirus recombinants in the West and East of China, 2) infected imported pangolins in the South, 3) bat sarbecovirus recombinant links to southwest of China, and 4) SARS-CoV-2 emergence toward Hubei in Central China (fig. 3*B*). Strikingly, the ranges of multiple species including *Rhinolophusaffinis*, *Rhinolophussinicus*, and *R. pusillus* overlap all the regions in China where nCoV members have been collected (fig. 4*B*). Other species known to harbor nCoV viruses have more restricted ranges such as *Rhinolophusmalayanus* found predominantly in the western part of China and countries to the Southwest of China (Myanmar, Thailand, Cambodia, Laos, Viet Nam, and Peninsular Malaysia) (Piraccini 2016; Bates et al. 2019). On the contrary, the greater horseshoe bat species, *Rhinolophusferrumequinum*, is not known to harbor any nCoV viruses and is absent from large parts of South Central China (fig. 4*B*).

The wide geographic ranges of *R. pusillus* and *R. affinis* and the fact that two of the closest known relatives of SARS-CoV-2, RpYN06, and RaTG13, have been sampled in these species flags them as prime suspects for the source of the SARS-CoV-2’s progenitor in China. Additionally, these two bat species are found in shared roosts with *R. sinicus* and *R. ferrumequinum* in Yunnan and with *R. sinicus* in Guangxi (Luo et al. 2013), providing opportunities for host switches, coinfections, and thus recombination between the sarbecoviruses that these bat species carry. *Rhinolophuspusillus* and *R. affinis* also link more regions of China with bat species such as *R. shameli*, *R. malayanus*, and *R. acuminatus* which are only found in Southeast Asia and southwest of China (fig. 4*B*). Latinne et al. (2020) published a large-scale sampling expedition of coronaviruses across bats in China. Despite there only being short RdRp sequence fragments available, the phylogeny for the novel viruses revealed a cluster of seven identical sarbecovirus sequences sampled from *R. affinis* within the nCoV clade (supplementary fig. S3, Supplementary Material online). Still, the fact that viruses in the Yunnan clade (consisting of RmYN02, RpYN06, and PrC31) were sampled from three different *Rhinolophus* species supports the hypothesis that these viruses readily infect multiple different horseshoe bat species with overlapping geographical ranges.

Based on the analysis of the sarbecovirus and host data presented here, we propose that to locate the SARS-CoV-2 progenitor sampling should focus on the ranges of horseshoe bat host populations known to harbor nCoV viruses. Specifically, samples should be collected in roosting environments spread across China with care taken both to avoid a further spillover (or reverse zoonosis) and to protect the bat populations (Luo et al. 2013). Sampling strategies will also need to consider the distinct subspecies of *Rhinolophus* as the delineators of genetically meaningful host populations for coronaviruses, for example, there are two *R. affinis* subspecies on mainland China: *himalayanus* and *macrurus* (Mao et al. 2010). Future sampling should also encompass a range of indigenous mammals other than bats that we now know can be infected by these coronaviruses. Although highly endangered, Chinese pangolins, given their susceptibility to infection and their geographical range across southern China (Challender et al. 2019), could be one of the possible candidates for the “missing” intermediate host of the SARS-CoV-2 proximal ancestor (WHO 2021).

## Conclusions

The currently available data, although sparse, illustrate a complex reticulate evolutionary history involving the lineage of sarbecoviruses SARS-CoV-2 emerged from. This history is governed by cocirculation of related coronaviruses, over at least the last 100 years, across the bat populations in southern China, and into Southeast Asia with multiple recombination events imprinted on the genomes of these viruses. Considering the high frequency of recombination, it is expected that selection could preferentially favor exchanges of specific genomic regions, in line with our detection of hotspots near the Spike gene (fig. 1*B* and *C*). The functional implications of selective Spike recombination has recently been corroborated by multiple independent studies, suggesting this might be a mechanism for antigenic shift utilized by the sarbecoviruses or, more broadly, by all coronavirus groups (Bobay et al. 2020; de Klerk et al. 2021; Goldstein et al. 2021; Nikolaidis et al. 2022; Yang et al. 2021). Our analysis further illustrates the importance of accounting for recombination rather than using whole-genome pairwise similarity to determine the shared evolutionary history of these viruses. This is exemplified by RaTG13 which is often described as the closest sarbecovirus to SARS-CoV-2 despite not being the phylogenetically closest virus once recombination history is accounted for in the other nCoV sarbecoviruses (figs. 1*A* and 3*A*).

The evidence of recombination events between members of the *Sarbecovirus* subgenus sampled in different geographical regions and from different bat hosts, indicates recent extensive movement of the viruses between different regions and species (and subspecies) as a result of the continued contacts between different bat populations that carry them. Although the closest known relatives of SARS-CoV-2 were sampled in Yunnan, the location of the proximal viral population SARS-CoV-2 emerged from remains unknown. The recombination patterns detected within the nCoV genomes imply the existence of one or several primary reservoir hosts with a geographical range spanning Thailand from the Southwest and Zhejiang to the East, a distribution that is consistent with specific Chinese horseshoe bats acting as the primary reservoir hosts. Our observations are further confirmed by a recent report of more bat coronaviruses very closely related to SARS-CoV-2 sampled from *R. pusillus* and *R. malayanus* in Laos (Temmam et al. 2022). Both the sampling location and host species are consistent with expectations based on our analysis, essentially filling in the geographic gap between previous nCoV sampling locations. The recombination patterns reported in these newly discovered genomes are also consistent with the extensive recombination reported here (Temmam et al. 2022). Having presented evidence in support of *R. affinis* and *R. pusillus’*s potential significance as the reservoir species, we would be remiss not to note that at least 20 different *Rhinolophus* species are distributed across China (four of which are endemic to China), many of which have not yet been found hosting nCoVs. The generalist nature of *Sarbecoviruses* also means multiple wild or farmed animals (e.g., American mink [*Neovison vison*] both farmed for fur and used as a food source) (WHO 2021; Xia et al. 2021; Xiao et al. 2021) could have facilitated transmission of SARS-CoV-2 from bats to humans.

Although SARS-like antibodies detected in people from rural communities in China (Wang et al. 2018; Li et al. 2019) indicates an intermediate animal species is potentially not required for transmission to humans, it does seem that emergence in a populated area is required for significant outbreaks to occur. The association of both SARS-CoV and SARS-CoV-2 with animal markets suggests animal trafficking and selling is a key part of this transmission to humans. Human-mediated animal movement increases contact with sarbecovirus infected animals (whether they are susceptible species that have been trapped or farmed in rural locations; Xia et al. 2021) and subsequently introduces them into city markets (Lytras et al. 2021; WHO 2021; Worobey 2021). Urgent questions relating to the prevention of another emergence are: where in China or Southeast Asia is the SARS-CoV-2 progenitor located (our analysis shows this is not necessarily Yunnan), which bat or other animal species are harboring nCoV sarbecoviruses and linked to this what is the risk of future spillover events? There is undoubtedly a virus highly related to SARS-CoV-2 still present somewhere in the wild. To maximize the probability that future sampling efforts will uncover this host species or subspecies we need a wide and systematic sampling strategy of horseshoe bats.

## Materials and Methods

### Genome Alignment

The whole-genome sequences of the 78 *Sarbecovirus* members used in this analysis (supplementary table S1, Supplementary Material online) were aligned and the ORF of the major protein-coding genes were defined based on SARS-CoV-2 annotation (Wu et al. 2020). Codon-level alignments of the ORFs were created using MAFFT v7.453 (Katoh and Standley 2013) and PAL2NAL (Suyama et al. 2006). The intergenic regions were also aligned separately using MAFFT and all alignments were pieced together into the final whole-genome alignment and visually inspected in Bioedit (Hall 1999).

### Genome-Specific Recombination Analysis

We first performed an analysis for detecting unique recombination events specific to individual genome sequences using the RDP (Martin and Rybicki 2000), GENECONV (Padidam et al. 1999), BOOTSCAN (Martin et al. 2005), MAXCHI (Smith 1992), CHIMAERA (Posada and Crandall 2001), SISCAN (Gibbs et al. 2000), and 3SEQ (Boni et al. 2007) methods implemented in the program RDP5 (Martin et al. 2021). Default settings were used throughout except: 1) only potential recombination events detected by three or more of the above methods, coupled with phylogenetic evidence of recombination were considered significant and 2) sequences were treated as linear. We required supporting evidence from three or more of the recombination signal detection methods because none of three methods query the same recombination signals and all have varying power to detect recombination in data sets with different degrees of sequence diversity (Posada and Crandall 2001; Posada 2002). The recombinant sequence identification, recombination breakpoint verification, and shared recombination event verification steps used are outlined in Martin et al. (2017). The approximate breakpoint positions and recombinant sequence(s) inferred for every potential recombination event, were manually checked and adjusted where necessary using the phylogenetic and recombination signal analysis features available in RDP5. Breakpoint positions were classified as undetermined if the 95% confidence interval on their location overlapped: 1) the 5′ and 3′ ends of the alignment; or 2) the position of a second detected breakpoint within the same sequence that had a lower associated *P* value (in such cases it could not be discounted that the actual breakpoint might not have simply been lost due to a more recent recombination event). All of the remaining breakpoint positions were manually checked and adjusted when necessary using the BURT method with the MAXCHI matrix and LARD two breakpoint scan methods (Holmes et al. 1999) used to resolve ties. A putatively nonrecombinant version of the original whole-genome alignment was reconstructed by excluding all minor parent sequence segments based on the supervised RDP5 analysis.

### Recombination Hotspot Analysis

The distribution of 236 unambiguously detected breakpoint positions defining 160 unique recombination events based on the RDP5 analysis described above were analyzed for evidence of recombination hotspots and coldspots using the permutation-based RRT (Simon-Loriere et al. 2009) and BDT (Heath et al. 2006). The RRT accounts for site-to-site variations in the detectability of individual recombination events and examines the distribution of point estimates of pairs of breakpoint locations bounding each of the unique recombination events detected by RDP5. Rather than using point estimates of recombination breakpoint locations, the BDT accounts for underlying uncertainties in the estimation of individual breakpoint locations as determined from the state transition likelihoods yielded by the hidden Markov model-based recombination breakpoint detection method, BURT (described in the RDP5 program manual at http://web.cbio.uct.ac.za/~darren/rdp.html).

To verify whether the recombination breakpoint clusters detected with these tests were consistent with the presence of recombination hotspots, we simulated recombination with SANTA-SIM (Jariani et al. 2019). Four data sets of 100×10 kb long sequences that had: 1) approximately the same degree of genetic diversity as the analyzed sarbecovirus alignment and 2) approximately the same numbers of detectable recombination events and recombination breakpoints per nucleotide as those detected in the analyzed sarbecovirus alignment. The SANTA-SIM settings used were: population size = 4,500, inoculum = all, mutation rate = 3.5×10^−5^, rate bias matrix = (0.42, 2.49, 0.29, 1.73, 0.23, 4.73, 6.99, 9.20, 0.60, 1.02, 2.56, 0.88), dual infection probability = 0.1, background recombination probability = 0.06, and generation number = 5,000. Simulated recombination events had a maximum of two breakpoints: a setting that required the use of a slightly modified version of SANTA-SIM that can be obtained from https://github.com/phillipswanepoel/santa-sim/tree/Recomb_and_align. Whereas one of the four data sets had no simulated recombination hotspots, the other three each had a single 100-nt-long hotspot between alignment positions 6000 and 6100 wherein recombination frequencies were 4×, 8×, or 16× higher than the background level.

All data sets were analyzed for recombination by RDP5 without any supervision, and RRT and BDT plots were produced for each data set (all with the same program settings used to analyze the actual sarbecovirus data set).

The true positive rate of the BDT was estimated as the proportion of 200-nt windows centered on nucleotides between positions 6000 and 6100, that is, within the simulated hotspot, that contained a number of breakpoints greater than the upper bound of the 99% confidence interval of the breakpoint clustering distribution expected under random recombination (e.g., indicated by the light gray areas of the plots in fig. 1*C*). Since a 200-nt sliding window was used for both breakpoint clustering tests, all windows overlapping with the hotspot (positions 5801 to positions 6299) were ignored when determining the BDT and RRT false positive rates. The false positive rate of BDT was calculated as the proportion (across all 100 simulated alignments of each of the four data sets) of the examined 200-nt windows centered on nucleotides outside region 5801–6299 that contained a number of breakpoints greater than the upper bound of the 99% confidence interval of the breakpoint clustering distribution expected under random recombination.

Similarly, the true positive rate of the RRT was estimated as the proportion, across all 100 simulated alignments in a data set, of 200-nt windows centered on nucleotides between positions 6000 and 6100, that is, within the simulated hotspot, that had associated breakpoint clustering permutation *P* values <0.01 (e.g., indicated by the upper bound of the light gray area of the plot in fig. 1*B*). The RRT false positive rate was calculated as the proportion, across all 100 simulated alignments in a data set, of the examined 200-nt windows centered on nucleotides outside region 5801–6339 that had associated permutation *P* values <0.01.

The true and false positive rates for BDT and RRT with respect to identifying the presence of the simulated recombination hotspots are indicated in supplementary table S2, Supplementary Material online. Note that, due to the nature of the simulations, it was not guaranteed that even with perfect recombination detection power and accuracy: 1) the recombination hotspot regions would contain any detectable excess of recombination breakpoints, and 2) the “normal” genome regions would contain no breakpoint clusters. What these simulations capture is the power of the two clustering tests to indirectly infer the locations of actual recombination hotspot regions that, due to chance during the simulation process, might not even contain any detectable recombination breakpoints. Nevertheless, as expected, the hotspot detection power of both BDT and RRT increases substantially with the intensity of the simulated recombination hotspots: from ∼10% for both tests with a 4× increase in simulated breakpoint probabilities within the 100-nt hotspot region to ∼60% for a 16× increase in breakpoint probabilities within the hotspot region. It is also noteworthy that the false positive rates for both tests are likely between 1.5 and 2× higher than the expected rate of 0.01 (which is expected given that the windows containing breakpoint clusters exceeding the 99% confidence interval were used to identify breakpoint hotspots). This false positive rate may not seem very high but, for a long alignment such as that examined for the sarbecoviruses that can be broken into ∼150 non-overlapping 200-nt windows, it indicates that for such an alignment we might expect to find on average two to three significant clusters of breakpoints that are in fact not associated with any elevation in the underlying recombination rate.

### Whole-Genome Alignment Recombination Analysis

Next, we sought to conservatively examine the entire genome alignment for the subset of recombination breakpoints that had the largest impacts on the inferred evolutionary relationships between the analyzed sarbecoviruses using the GARD method (Kosakovsky et al. 2006) implemented in Hyphy v2.5.29 (Kosakovsky Pond et al. 2020). Model goodness of fit was evaluated using the small sample Akaike Inference Criterion (c-AIC) (Akaike 1998). To improve computational efficiency and statistical efficiency (as GARD requires more statistical evidence of recombination for larger phylogenies, and the minimal length of detectable nonrecombinant fragments increases with the number of sequences) and focus on the closest relatives of SARS-CoV-2, 22 of the 78 viruses that are closest to SARS-CoV-2 or had preliminary evidence of clustering near detected interclade recombinants were included in the GARD analysis (supplementary table S1, Supplementary Material online). Only breakpoints present in more than 2/3 of the 64 GARD consecutive step-up models were retained to produce a final set of 21 likely breakpoints (positions corresponding to the SARS-CoV-2 reference genome Wuhan-Hu-1 in order: 1680, 3093, 3649, 4973, 8208, 11445, 12622, 14401, 15954, 16923, 19965, 20518, 21198, 21411, 22460, 23396, 24144, 24843, 26323, 27388, 27685). Based on these the whole-genome alignment was split into 22 RBP regions. The position of each region on the alignment and relative to the SARS-CoV-2 genome as well as their length is presented in supplementary table S3, Supplementary Material online.

We further used the GARD recombination analysis to validate the RDP5 recombination hotspot analysis. We performed a permutation test of breakpoint clustering by fixing the number of all inferred breakpoints (64) and the location of the 13,550 variable sites in the alignment. Then defined a sliding window so that each window would have an average of one breakpoint in it (alignment length/64) producing 474 windows. *N* = 10,000 replicates were drawn where 64 variable sites were randomly chosen from one of the breakpoints. For each sliding window, we tabulated the distribution of randomly drawn breakpoints in the window. Two hotspots and 17 coldspot windows were identified, presented in supplementary figure S2, Supplementary Material online. This analysis is not expected to produce results identical to the RDP5-based hotspot analysis, since the GARD method does not distinguish between potential breakpoints in very near genomic proximity, so this post hoc test is unlikely to identify clustering of unique breakpoints that are very close to one another (in contrast to the RDP5 approach).

### Phylogenetic Reconstruction

The phylogeny of each RBP alignment region based on the GARD analysis and the nonrecombinant whole-genome based on the RDP5 analysis were reconstructed using iqtree version 1.6.12 (Nguyen et al. 2015) under a general time reversible (GTR) substitution model assuming invariable sites and a four-category Γ distribution. Tree node confidence was determined using 10,000 ultrafast bootstrap replicates.

Based on the nonrecombinant whole-genome phylogeny, 20 viruses form a monophyletic nCoV clade (fig. 1*A*). To illustrate the distance of each virus from SARS-CoV-2 for each GARD determined genomic region, we defined the nCoV clade on each phylogeny as the subset of the aforementioned 20 nCoV viruses forming a monophyly with SARS-CoV-2 in each phylogeny. The rest of the viruses were classified as members of the non-nCoV clade for each RBP region. We then used an arbitrary tip distance scale normalized between all phylogenies so distances are comparable between regions. For each maximum likelihood tree, the patristic distance between each tip and SARS-CoV-2 is calculated using ETE 3 (Huerta-Cepas et al. 2016) as d1 for members of the nCoV clade and d2 for members of the non-nCoV clade. The distances are then normalized so that for nCoV clade members range between 0.1 and 1.1 (1.1 being SARS-CoV-2 itself and 0.1 being the most distant tip from SARS-CoV-2 within the nCoV clade) and between −0.1 and −1.1 for non-nCoV members (−0.1 being the closest non-nCoV virus to SARS-CoV-2 and −1.1 the most distant), as follows:

$${d'}_{1}=1.1-\frac{d_{1}}{d_{1,\max}}(1:\mathrm{nCoV})$$

$${d'}_{2}=-0.1-\frac{d_{2}-d_{2,\min}}{d_{2,\max}-d_{2,\min}}(2:\mathrm{non}-\mathrm{nCoV}).$$

With *d* ′_1_ and *d* ′_2_ being the normalized values for each clade, variables denoted with “min” being the smallest distance and variables denoted with “max” being the largest distance in each given set.

Phylogenies were visualized using FigTree (http://tree.bio.ed.ac.uk/software/figtree/) and ETE 3 (Huerta-Cepas et al. 2016).

### Molecular Dating

To provide temporal information to the phylogenetic history of the viruses, we performed a Bayesian phylogenetic analysis on RBP region 5, using BEAST v1.10.4 (Suchard et al. 2018). This region was selected due to its length, being one of the two longest nonrecombinant regions in the analysis (3,238 bp), and because all 20 nCoV viruses form a monophyly in the respective tree. Based on the observation of an increased evolutionary rate specific to the deepest branch of the nCoV clade reported in MacLean et al. (2021) (MacLean et al. 2021), we adopted the same approach of fitting a separate local clock model to that branch from the rest of the phylogeny. A normal rate distribution with mean 5×10^−4^ and SD 2×10^−4^ was used as an informative prior on all other branches. The lineage containing the BtKY72 and BM48-31 bat viruses was constrained as the outgroup to maintain overall topology. Codon positions were partitioned and a GTR + Γ substitution model was specified independently for each partition. The maximum likelihood phylogeny reconstructed previously for RBP region 5 was used as a starting tree (rooted at the BtKY72 and BM48-31 clade). A constant size coalescent model was used for the tree prior and a lognormal prior with a mean of 6 and SD of 0.5 was specified on the population size. Two independent MCMC runs were performed for 500 million states for the data set. The two chains were inspected for convergence and combined using LogCombiner (Drummond and Rambaut 2007) using a 10% burn-in for each chain. The effective sample size for all estimated parameters was above 200.

### Host Range Data

All host ranges presented in figure 4*B* were retrieved from the IUCN Red List of Threatened Species (https://www.iucnredlist.org/) and the Mammals of China (Princeton Pocket Guide) (Hoffmann et al. 2013). Geographic visualization was performed using D3 and JavaScript in Observable (https://observablehq.com/). 

## Supplementary Material

Supplementary data are available at *Genome Biology and Evolution* online.

## Supplementary Material

evac018_Supplementary_Data

## References

[evac018-B1] Akaike H. 1998. Information theory and an extension of the maximum likelihood principle. In: Parzen E, Tanabe K, Kitagawa G, editors. Selected Papers of Hirotugu Akaike. Springer Series in Statistics (Perspectives in Statistics). New York: Springer. p. 199–213.

[evac018-B2] Bates P , Bumrungsri S, Csorba G, Soisook P. 2019. *Rhinolophus malayanus*. IUCN Red List Threat. Species 2019. [Internet]:e.T19551A21978424. International Union for Conservation of Nature and Natural Resources (IUCN). Available from: https://www.iucnredlist.org/species/19551/21978424.

[evac018-B3] Bobay LM , O’Donnell AC, Ochman H. 2020. Recombination events are concentrated in the spike protein region of Betacoronaviruses. PLoS Genet. 16(12):e1009272. 33332358 10.1371/journal.pgen.1009272 PMC7775116

[evac018-B4] Boni MF , et al 2020. Evolutionary origins of the SARS-CoV-2 sarbecovirus lineage responsible for the COVID-19 pandemic. Nat Microbiol. 5(11):1408–1417. 32724171 10.1038/s41564-020-0771-4

[evac018-B5] Boni MF , Posada D, Feldman MW. 2007. An exact nonparametric method for inferring mosaic structure in sequence triplets. Genetics 176(2):1035–1047. 17409078 10.1534/genetics.106.068874 PMC1894573

[evac018-B6] Challender D , et al 2019. *Manis pentadactyla.* IUCN Red List Threat. Species 2019. [Internet]:e.T12764A168392151. International Union for Conservation of Nature and Natural Resources (IUCN). Available from: https://www.iucnredlist.org/species/12764/168392151.

[evac018-B7] Conceicao C , et al 2020. The SARS-CoV-2 spike protein has a broad tropism for mammalian ACE2 proteins. PLoS Biol. 18(12):e3001016. 33347434 10.1371/journal.pbio.3001016 PMC7751883

[evac018-B8] de Klerk A , et al 2021. Conserved recombination patterns across coronavirus subgenera. *bioRxiv* [Internet]:2021.11.21.469423. Available from: https://www.biorxiv.org/content/10.1101/2021.11.21.469423v1. 10.1093/ve/veac054 PMC9261289 35814334

[evac018-B9] Delaune D , et al 2021. A novel SARS-CoV-2 related coronavirus in bats from Cambodia. Nat Commun. 12:1–7. 34753934 10.1038/s41467-021-26809-4 PMC8578604

[evac018-B10] Drummond AJ , Rambaut A. 2007. BEAST: Bayesian evolutionary analysis by sampling trees. BMC Evol Biol. 7:1–8. 17996036 10.1186/1471-2148-7-214 PMC2247476

[evac018-B11] Gibbs MJ , Armstrong JS, Gibbs AJ. 2000. Sister-scanning: a Monte Carlo procedure for assessing signals in recombinant sequences. Bioinformatics 16(7):573–582. 11038328 10.1093/bioinformatics/16.7.573

[evac018-B12] Goldstein SA , Brown J, Pedersen BS, Quinlan AR, Elde NC. 2021. Extensive recombination-driven coronavirus diversification expands the pool of potential pandemic pathogens. *bioRxiv* [Internet]:2021.02.03.429646. Available from: https://www.biorxiv.org/content/10.1101/2021.02.03.429646v2. 10.1093/gbe/evac161 PMC9730504 36477201

[evac018-B13] Gorbalenya AE , et al 2020. The species Severe acute respiratory syndrome-related coronavirus: classifying 2019-nCoV and naming it SARS-CoV-2. Nat Microbiol. 5:536–544. 32123347 10.1038/s41564-020-0695-z PMC7095448

[evac018-B14] Graham RL , Baric RS. 2010. Recombination, reservoirs, and the modular spike: mechanisms of coronavirus cross-species transmission. J Virol. 84(7):3134–3146. 19906932 10.1128/JVI.01394-09 PMC2838128

[evac018-B15] Hall TA. 1999. BioEdit a user-friendly biological sequence alignment editor and analysis program for Windows 95/98/NT. In: Nucleic Acids Symposium Series 41. p. 95–98. Oxford: Oxford University Press.

[evac018-B16] Heath L , van der Walt E, Varsani A, Martin DP. 2006. Recombination patterns in aphthoviruses mirror those found in other Picornaviruses. J Virol. 80(23):11827–11832. 16971423 10.1128/JVI.01100-06 PMC1642601

[evac018-B17] Hoffmann RS , Lunde D, MacKinnon J, Wilson DE, Wozencraft WC. 2013. Princeton pocket guides: mammals of China. Oxfordshire: Princeton University Press.

[evac018-B18] Holmes EC , et al 2021. The origins of SARS-CoV-2: a critical review. Cell 184(19):4848–4856. 34480864 10.1016/j.cell.2021.08.017 PMC8373617

[evac018-B19] Holmes EC , Worobey M, Rambaut A. 1999. Phylogenetic evidence for recombination in dengue virus. Mol Biol Evol. 16(3):405–409. 10331266 10.1093/oxfordjournals.molbev.a026121

[evac018-B20] Hu D , et al 2018. Genomic characterization and infectivity of a novel SARS-like coronavirus in Chinese bats. Emerg Microbes Infect. 7(1):1. 30209269 10.1038/s41426-018-0155-5 PMC6135831

[evac018-B21] Huerta-Cepas J , Serra F, Bork P. 2016. ETE 3: reconstruction, analysis, and visualization of phylogenomic data. Mol Biol Evol. 33(6):1635–1638. 26921390 10.1093/molbev/msw046 PMC4868116

[evac018-B22] Jackson B , et al 2021. Generation and transmission of interlineage recombinants in the SARS-CoV-2 pandemic. Cell 184(20):5179–5188*.* 10.1016/j.cell.2021.08.014 PMC8367733 34499854

[evac018-B23] Jariani A , et al 2019. SANTA-SIM: simulating viral sequence evolution dynamics under selection and recombination. Virus Evol. 5:vez003. 30863552 10.1093/ve/vez003 PMC6407609

[evac018-B24] Katoh K , Standley DM. 2013. MAFFT Multiple Sequence Alignment Software version 7: improvements in performance and usability. Mol Biol Evol. 30(4):772–780. 23329690 10.1093/molbev/mst010 PMC3603318

[evac018-B25] Kosakovsky Pond SL , et al 2020. HyPhy 2.5-A customizable platform for evolutionary hypothesis testing using phylogenies. Mol Biol Evol. 37(1):295–299. 31504749 10.1093/molbev/msz197 PMC8204705

[evac018-B26] Kosakovsky SL , Posada D, Gravenor MB, Woelk CH, Frost SDW. 2006. GARD: a genetic algorithm for recombination detection. Bioinformatics 22(24):3096–3098. 17110367 10.1093/bioinformatics/btl474

[evac018-B27] Lam TTY , et al 2020. Identifying SARS-CoV-2 related coronaviruses in Malayan pangolins. Nature 583(7815):282–285. 32218527 10.1038/s41586-020-2169-0

[evac018-B28] Latinne A , et al 2020. Origin and cross-species transmission of bat coronaviruses in China. Nat Commun. 11:1–15. 32843626 10.1038/s41467-020-17687-3 PMC7447761

[evac018-B29] Lee J , et al 2020. No evidence of coronaviruses or other potentially zoonotic viruses in Sunda pangolins (*Manis javanica*) entering the wildlife trade via Malaysia. Ecohealth 17(3):406–418. 33226526 10.1007/s10393-020-01503-x PMC7682123

[evac018-B30] Li H , et al 2019. Human-animal interactions and bat coronavirus spillover potential among rural residents in Southern China. Biosaf Health. 1(2):84–90. 32501444 10.1016/j.bsheal.2019.10.004 PMC7148670

[evac018-B31] Li L , et al 2021. A novel SARS-CoV-2 related coronavirus with complex recombination isolated from bats in Yunnan province, China. Emerg Microbes Infect. 10(1):1683–1690. 34348599 10.1080/22221751.2021.1964925 PMC8381922

[evac018-B32] Li Q , Guan X, et al 2020. Early transmission dynamics in Wuhan, China, of novel coronavirus–infected pneumonia. N Engl J Med. 382(13):1199–1207. 31995857 10.1056/NEJMoa2001316 PMC7121484

[evac018-B33] Li X , Giorgi EE, et al 2020. Emergence of SARS-CoV-2 through recombination and strong purifying selection. Sci Adv. 6(27):eabb9153. 32937441 10.1126/sciadv.abb9153 PMC7458444

[evac018-B34] Lin XD , et al 2017. Extensive diversity of coronaviruses in bats from China. Virology 507:1–10. 28384506 10.1016/j.virol.2017.03.019 PMC7111643

[evac018-B35] Liu P , Chen W, Chen J-P. 2019. Viral metagenomics revealed Sendai virus and coronavirus infection of Malayan Pangolins (*Manis javanica*). Viruses 11(11):979. 10.3390/v11110979 PMC6893680 31652964

[evac018-B36] Luo J , et al 2013. Bat conservation in China: should protection of subterranean habitats be a priority? ORYX 47(4):526–531.

[evac018-B37] Lytras S , Xia W, Hughes J, Jiang X, Robertson DL. 2021. The animal origin of SARS-CoV-2. Science 373(6558):968–970. 34404734 10.1126/science.abh0117

[evac018-B38] MacLean OA , et al 2021. Natural selection in the evolution of SARS-CoV-2 in bats created a generalist virus and highly capable human pathogen. PLoS Biol. 19(3):e3001115. 33711012 10.1371/journal.pbio.3001115 PMC7990310

[evac018-B39] Mao XG , Zhu GJ, Zhang S, Rossiter SJ. 2010. Pleistocene climatic cycling drives intra-specific diversification in the intermediate horseshoe bat (*Rhinolophus affinis*) in Southern China. Mol Ecol. 19(13):2754–2769. 20561192 10.1111/j.1365-294X.2010.04704.x

[evac018-B40] Martin D , Rybicki E. 2000. RDP: detection of recombination amongst aligned sequences. Bioinformatics 16(6):562–563. 10.1093/bioinformatics/16.6.562 10980155

[evac018-B41] Martin DP , et al 2021. RDP5: a computer program for analysing recombination in, and removing signals of recombination from, nucleotide sequence datasets. Virus Evol. 7:87. 10.1093/ve/veaa087 PMC8062008 33936774

[evac018-B42] Martin DP , Murrell B, Khoosal A, Muhire B. 2017. Detecting and analyzing genetic recombination using RDP4. In: Keith JM, editor. Methods in molecular biology. Vol. 1525. Totowa (NJ): Humana Press Inc. p. 433–460. 27896731 10.1007/978-1-4939-6622-6_17

[evac018-B43] Martin DP , Posada D, Crandall KA, Williamson C. 2005. A modified bootscan algorithm for automated identification of recombinant sequences and recombination breakpoints. AIDS Res Hum Retroviruses. 21(1):98–102. 15665649 10.1089/aid.2005.21.98

[evac018-B44] Nguyen LT , Schmidt HA, Von Haeseler A, Minh BQ. 2015. IQ-TREE: a fast and effective stochastic algorithm for estimating maximum-likelihood phylogenies. Mol Biol Evol. 32(1):268–274. 25371430 10.1093/molbev/msu300 PMC4271533

[evac018-B45] Nikolaidis M , Markoulatos P, Van de Peer Y, Oliver SG, Amoutzias GD. 2022. The neighborhood of the spike gene is a hotspot for modular intertypic homologous and nonhomologous recombination in coronavirus genomes. Mol Biol Evol. 39(1):msab292. 10.1093/molbev/msab292 PMC8549283 34638137

[evac018-B46] Oude Munnink BB , et al 2021. Transmission of SARS-CoV-2 on mink farms between humans and mink and back to humans. Science 371(6525):172–177. 33172935 10.1126/science.abe5901 PMC7857398

[evac018-B47] Padidam M , Sawyer S, Fauquet CM. 1999. Possible emergence of new geminiviruses by frequent recombination. Virology 265(2):218–225. 10600594 10.1006/viro.1999.0056

[evac018-B48] Piraccini R. 2016. *Rhinolophus ferrumequinum*. IUCN Red List Threat. Species 2016. [Internet]:e.T19517A21973253. International Union for Conservation of Nature and Natural Resources (IUCN).

[evac018-B49] Posada D. 2002. Evaluation of methods for detecting recombination from DNA sequences: empirical data. Mol Biol Evol. 19(5):708–717. 11961104 10.1093/oxfordjournals.molbev.a004129

[evac018-B50] Posada D , Crandall KA. 2001. Evaluation of methods for detecting recombination from DNA sequences: computer simulations. Proc Natl Acad Sci U S A. 98(24):13757–13762. 11717435 10.1073/pnas.241370698 PMC61114

[evac018-B51] Simon-Loriere E , et al 2009. Molecular mechanisms of recombination restriction in the envelope gene of the human immunodeficiency virus. PLoS Pathog. 5(5):e1000418. 19424420 10.1371/journal.ppat.1000418 PMC2671596

[evac018-B52] Smith JM. 1992. Analyzing the mosaic structure of genes. J Mol Evol. 34(2):126–129. 1556748 10.1007/BF00182389

[evac018-B53] Sola I , Almazán F, Zúñiga S, Enjuanes L. 2015. Continuous and discontinuous RNA synthesis in coronaviruses. Annu Rev Virol. 2(1):265–288. 26958916 10.1146/annurev-virology-100114-055218 PMC6025776

[evac018-B54] Suchard MA , et al 2018. Bayesian phylogenetic and phylodynamic data integration using BEAST 1.10. Virus Evol. 4(1):vey016. 29942656 10.1093/ve/vey016 PMC6007674

[evac018-B55] Suyama M , Torrents D, Bork P. 2006. PAL2NAL: robust conversion of protein sequence alignments into the corresponding codon alignments. Nucleic Acids Res. 34(Web Server issue):W609–W612. 16845082 10.1093/nar/gkl315 PMC1538804

[evac018-B56] Temmam S , et al 2022. Bat coronaviruses related to SARS-CoV-2 and infectious for human cells. Nature. Available from: https:// doi.org/10.1038/s41586-022-04532-4. 10.1038/s41586-022-04532-4 35172323

[evac018-B57] Wacharapluesadee S , et al 2021. Evidence for SARS-CoV-2 related coronaviruses circulating in bats and pangolins in Southeast Asia. Nat Commun. 12:972. 33563978 10.1038/s41467-021-21240-1 PMC7873279

[evac018-B58] Walls AC , et al 2020. Structure, function, and antigenicity of the SARS-CoV-2 spike glycoprotein. Cell 181(2):281–292.e6. 32155444 10.1016/j.cell.2020.02.058 PMC7102599

[evac018-B59] Wang H , Pipes L, Nielsen R. 2021. Synonymous mutations and the molecular evolution of SARS-CoV-2 origins. Virus Evol. 7:veaa098. 33500788 10.1093/ve/veaa098 PMC7798566

[evac018-B60] Wang N , et al 2018. Serological evidence of bat SARS-related coronavirus infection in humans, China. Virol Sin. 33(1):104–107. 29500691 10.1007/s12250-018-0012-7 PMC6178078

[evac018-B61] Wells HL , et al 2021. The evolutionary history of ACE2 usage within the coronavirus subgenus Sarbecovirus. Virus Evol. 7(1):veab007. 33754082 10.1093/ve/veab007 PMC7928622

[evac018-B62] WHO. 2021. WHO-convened global study of origins of SARS-CoV-2. WHO. Available from: https://www.who.int/publications/i/item/who-convened-global-study-of-origins-of-sars-cov-2-china-part.

[evac018-B63] Worobey M. 2021. Dissecting the early COVID-19 cases in Wuhan. Science 374(6572):1202–1204. 34793199 10.1126/science.abm4454

[evac018-B64] Wu F , et al 2020. A new coronavirus associated with human respiratory disease in China. Nature 579(7798):265–269. 32015508 10.1038/s41586-020-2008-3 PMC7094943

[evac018-B65] Xia W , Hughes J, Robertson DL, Jiang X. 2021. How one pandemic led to another: ASFV, the disruption contributing to SARS-CoV-2 emergence in Wuhan. Preprints [Internet]. Available from: https://www.preprints.org/manuscript/202102.0590/v1.

[evac018-B66] Xiao K , et al 2020. Isolation of SARS-CoV-2-related coronavirus from Malayan pangolins. Nature 583(7815):286–289. 32380510 10.1038/s41586-020-2313-x

[evac018-B67] Xiao X , Newman C, Buesching CD, Macdonald DW, Zhou Z-M. 2021. Animal sales from Wuhan wet markets immediately prior to the COVID-19 pandemic. Sci Rep. 11:1–7. 34099828 10.1038/s41598-021-91470-2 PMC8184983

[evac018-B68] Xu L , Guan J, Lau W, Xiao Y. 2016. An overview of pangolin trade in China – Wildlife Trade Report from TRAFFIC. Traffic [Internet]. Available from: https://www.traffic.org/publications/reports/pangolin-trade-in-china/.

[evac018-B69] Yang Y , Yan W, Hall AB, Jiang X. 2021. Characterizing transcriptional regulatory sequences in coronaviruses and their role in recombination. Mol Biol Evol. 38(4):1241–1248. 33146390 10.1093/molbev/msaa281 PMC7665640

[evac018-B70] Zhou H , Chen X, Hughes AC, Bi Y, Shi W. 2020. A novel bat coronavirus closely related to SARS-CoV-2 contains natural insertions at the S1/S2 cleavage site of the spike protein. Curr Biol. 30:1–8. 33022232 10.1016/j.cub.2020.09.030 PMC7534657

[evac018-B71] Zhou H , et al 2021. Identification of novel bat coronaviruses sheds light on the evolutionary origins of SARS-CoV-2 and related viruses. Cell 184(17):4380–4312. 34147139 10.1016/j.cell.2021.06.008 PMC8188299

[evac018-B72] Zhou P , Yang X-L, et al 2020. A pneumonia outbreak associated with a new coronavirus of probable bat origin. Nature 579(7798):270–273. 32015507 10.1038/s41586-020-2012-7 PMC7095418

