# Supplementary material for: Exploring the Natural Origins of SARS-CoV-2 in the Light of Recombination
Source: Genome Biol Evol. 2022 Feb 8;14(2):evac018. doi: 10.1093/gbe/evac018 (PMC8882382; doi:10.1093/gbe/evac018)
Supplement: evac018_Supplementary_Data [file evac018_supplementary_data.zip › Figures_S1toS3.pdf]

Exploring the natural origins of SARS-CoV-2 in the light of recombination
Supplementary Figures

Spyros Lytras[1], Joseph Hughes[1], Darren Martin[2], Phillip Swanepoel [2], Arné de Klerk[2], Rentia Lourens[3],Sergei Kosakovsky Pond[4], Wei Xia[5], Xiaowei Jiang[6], David L Robertson[1]

[1]*MRC-University of Glasgow Centre for Virus Research, Glasgow, UK.*
[2]*Computational Biology Division, Department of Integrative Biomedical Sciences, University of Cape Town, Cape Town, South Africa.*
[3]*Division of Neurosurgery, Department of Surgery, Neuroscience institute, University of Cape Town, Cape Town, South Africa.*
[4]*Institute for Genomics and Evolutionary Medicine, Department of Biology, Temple University, Pennsylvania, USA.*
[5]*National School of Agricultural Institution and Development, South China Agricultural University, Guangzhou, China.*
[6]*Department of Biological Sciences, Xi'an Jiaotong-Liverpool University (XJTLU), Suzhou, China.*

1 Figure S1

Figure S1. Maximum likelihood phylogenies for all 22 recombination breakpoint partitioned (RBP) regions of the analysis. The nCoV clade is annotated in pink and the non-nCoV clade in blue. SARS-CoV-2 and SARS-CoV are highlighted in pink and blue respectively. Branch length (top) and bootstrap support (bottom) are shown on every node.

RBP region 1

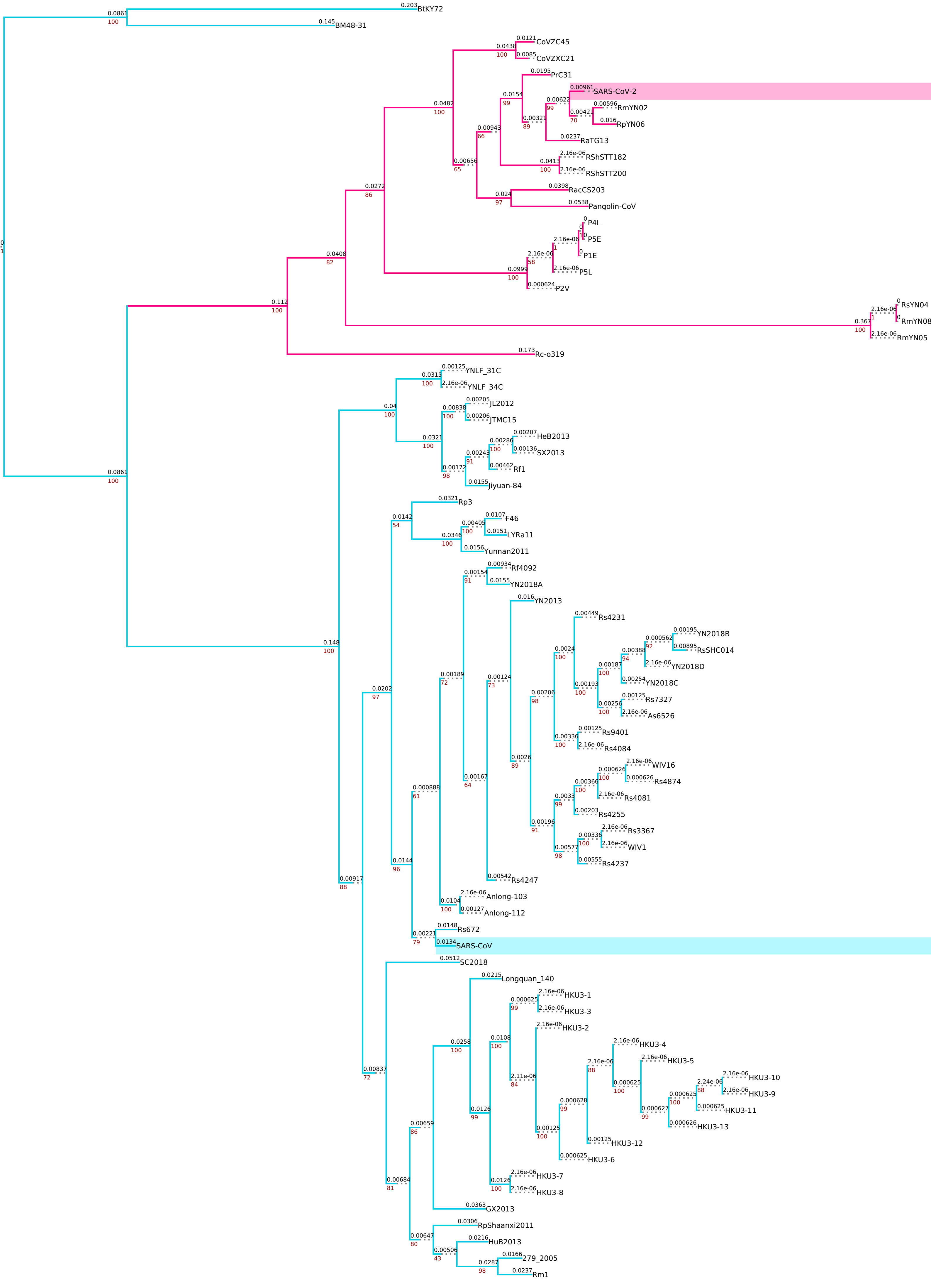

RBP region 2

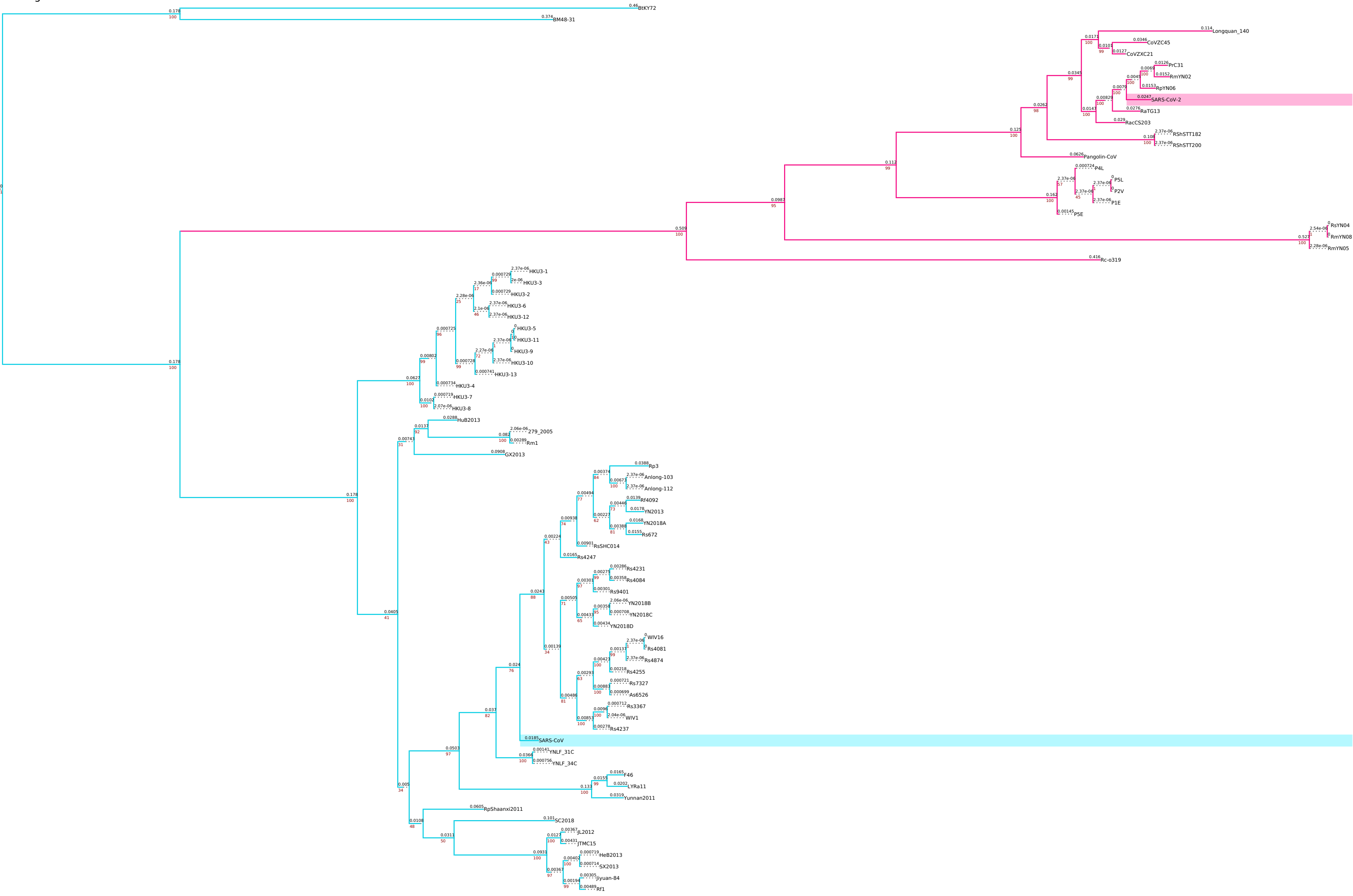
BtKY72
BM48-31
Longquan_140
CoVZC45
CoVZXC21
PrC31
RmYN02
RpYN06
SARS-CoV-2
RaTG13
PacCS203
RShSTT182
RShSTT200
Pangolin-CoV
P4L
P5L
P2V
P1E
P5E
RsYN04
RmYN08
RmYN05
Rc-o319
HKU3-1
HKU3-3
HKU3-2
HKU3-6
HKU3-12
HKU3-5
HKU3-11
HKU3-9
HKU3-10
HKU3-13
HKU3-4
HKU3-7
HKU3-8
HuB2013
279_2005
Rm1
GX2013
Rp3
Anlong-103
Anlong-112
Rf4092
YN2013
YN2018A
Rs672
RsSHC014
Rs4247
Rs4231
Rs4084
Rs9401
YN2018B
YN2018C
YN2018D
WIV16
Rs4081
Rs4874
Rs4255
Rs7327
As6526
Rs3367
WIV1
Rs4237
SARS-CoV
YNLF_31C
YNLF_34C
F46
LYRa11
Yunnan2011
RpShaanxi2011
SC2018
JL2012
JTMC15
HeB2013
SX2013
Jiyuan-84
Rf1

RBP region 3

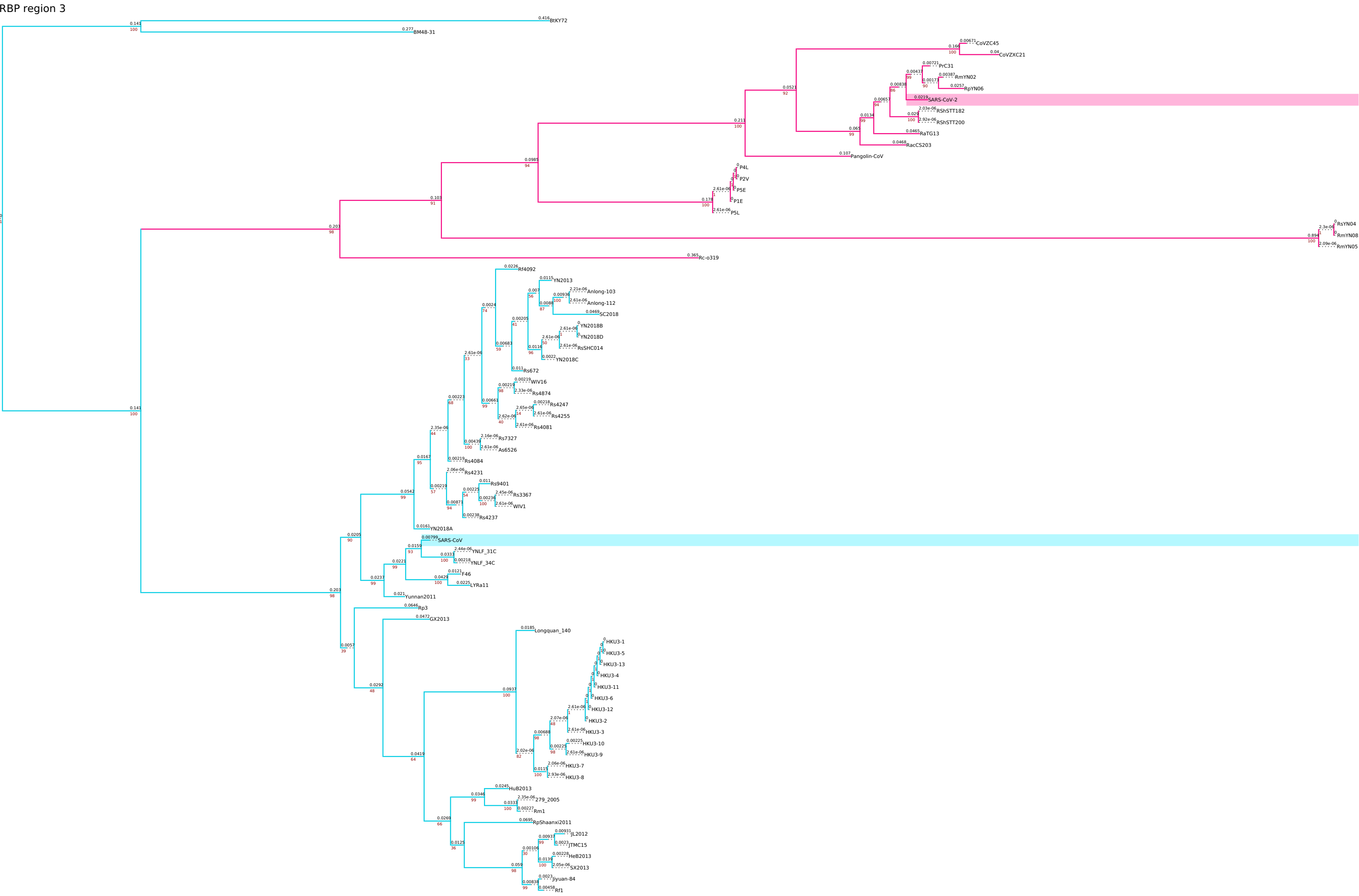
BtKY72
BM48-31
CoVZC45
CoVZXC21
PrC31
RmYN02
RpYN06
SARS-CoV-2
RShSTT182
RShSTT200
RaTG13
RacCS203
Pangolin-CoV
P4L
P2V
P5E
P1E
P5L
RsYN04
RmYN08
RmYN05
Rc-o319
Rf4092
YN2013
Anlong-103
Anlong-112
SC2018
YN2018B
YN2018D
RsSHC014
YN2018C
Rs672
WIV16
Rs4874
Rs4247
Rs4255
Rs4081
Rs7327
As6526
Rs4084
Rs4231
Rs9401
Rs3367
WIV1
Rs4237
YN2018A
SARS-CoV
YNLF_31C
YNLF_34C
F46
LYRa11
Yunnan2011
Rp3
GX2013
Longquan_140
HKU3-1
HKU3-5
HKU3-13
HKU3-4
HKU3-11
HKU3-6
HKU3-12
HKU3-2
HKU3-3
HKU3-10
HKU3-9
HKU3-7
HKU3-8
HuB2013
279_2005
Rm1
RpShaanxi2011
JL2012
JTMC15
HeB2013
SX2013
Jiyuan-84
Rf1

RBP region 4

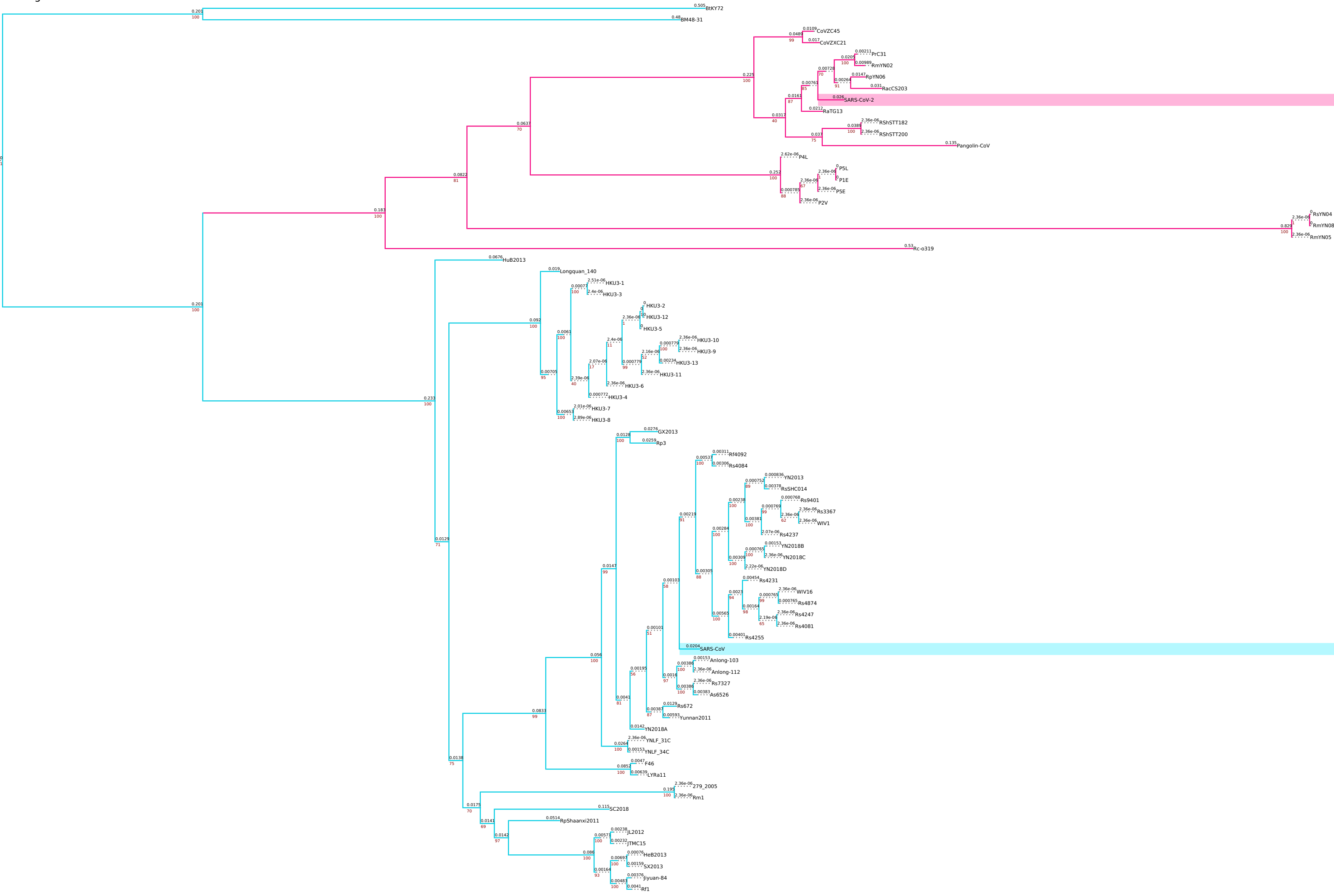

BtKY72
BM48-31
CoVZC45
CoVZXC21
PrC31
RmYN02
RpYN06
RacCS203
SARS-CoV-2
RaTG13
RShSTT182
RShSTT200
Pangolin-CoV
P4L
P5L
P1E
P5E
P2V
RsYN04
RmYN08
RmYN05
Rc-o319
HuB2013
Longquan_140
HKU3-1
HKU3-3
HKU3-2
HKU3-12
HKU3-5
HKU3-10
HKU3-9
HKU3-13
HKU3-11
HKU3-6
HKU3-4
HKU3-7
HKU3-8
GX2013
Rp3
Rf4092
Rs4084
YN2013
RsSHC014
Rs9401
Rs3367
WIV1
Rs4237
YN2018B
YN2018C
YN2018D
Rs4231
WIV16
Rs4874
Rs4247
Rs4081
Rs4255
SARS-CoV
Anlong-103
Anlong-112
Rs7327
As6526
Rs672
Yunnan2011
YN2018A
YNLF_31C
YNLF_34C
F46
LYRa11
279_2005
Rm1
SC2018
RpShaanxi2011
JL2012
JTMC15
HeB2013
SX2013
Jiyuan-84
Rf1

RBP region 5

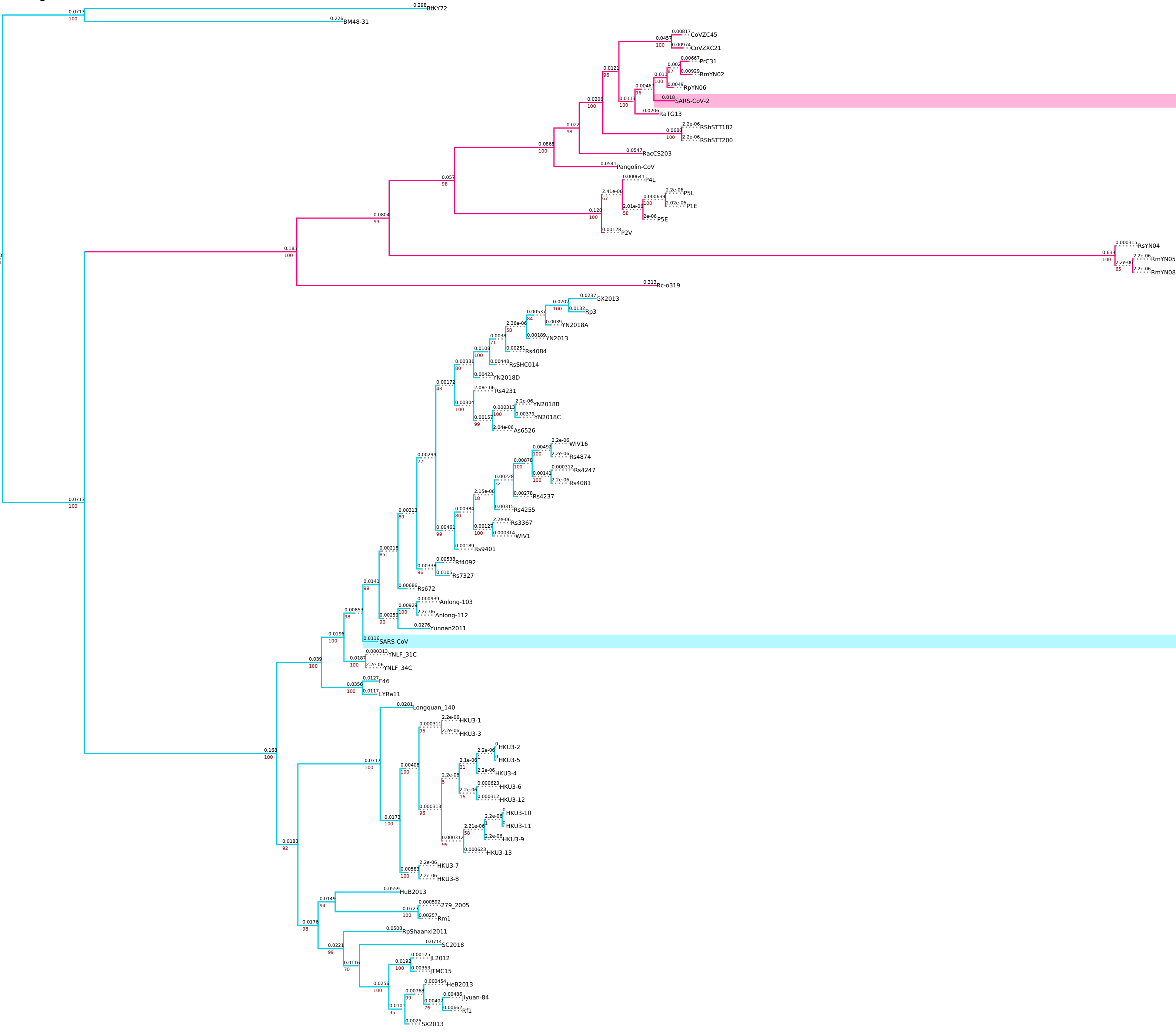

BtKY72
BM48-31
CoVZC45
CoVZXC21
PrC31
RmYN02
RpYN06
SARS-CoV-2
RaTG13
RShSTT182
RShSTT200
RacCS203
Pangolin-CoV
P4L
P5L
P1E
P5E
P2V
RsYN04
RmYN05
RmYN08
Rc-o319
GX2013
Rp3
YN2018A
YN2013
Rs4084
RsSHC014
YN2018D
Rs4231
YN2018B
YN2018C
As6526
WIV16
Rs4874
Rs4247
Rs4081
Rs4237
Rs4255
Rs3367
WIV1
Rs9401
Rf4092
Rs7327
Rs672
Anlong-103
Anlong-112
Yunnan2011
SARS-CoV
YNLF_31C
YNLF_34C
F46
LYRa11
Longquan_140
HKU3-1
HKU3-3
HKU3-2
HKU3-5
HKU3-4
HKU3-6
HKU3-12
HKU3-10
HKU3-11
HKU3-9
HKU3-13
HKU3-7
HKU3-8
HuB2013
279_2005
Rm1
RpShaanxi2011
SC2018
JL2012
JTMC15
HeB2013
Jiyuan-84
Rf1
SX2013

RBP region 6

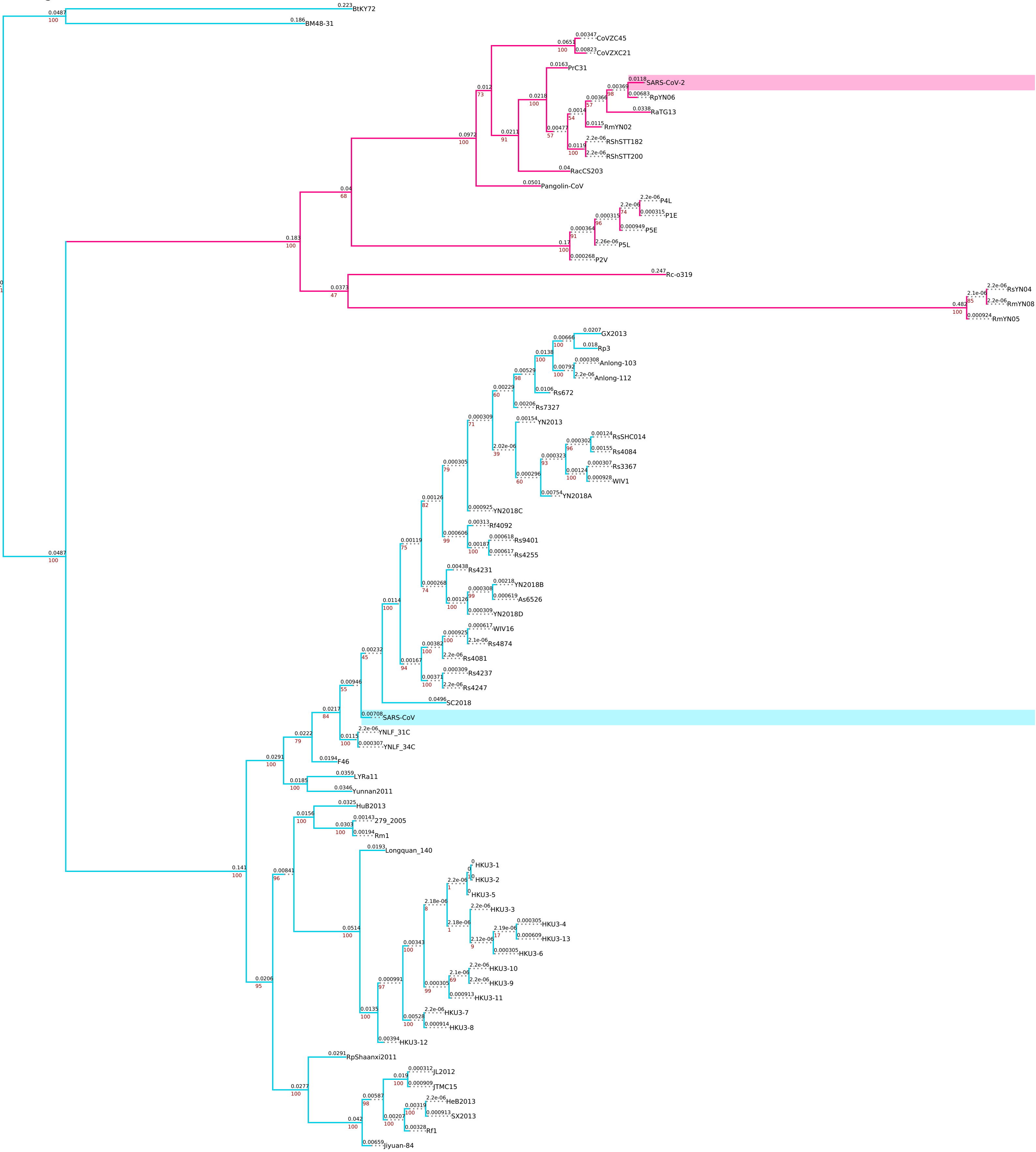

BtKY72
BM48-31
CoVZC45
CoVZXC21
PrC31
SARS-CoV-2
RpYN06
RaTG13
RmYN02
RShSTT182
RShSTT200
RacCS203
Pangolin-CoV
P4L
P1E
P5E
P5L
P2V
Rc-o319
RsYN04
RmYN08
RmYN05
GX2013
Rp3
Anlong-103
Anlong-112
Rs672
Rs7327
YN2013
RsSHC014
Rs4084
Rs3367
WIV1
YN2018A
YN2018C
Rf4092
Rs9401
Rs4255
Rs4231
YN2018B
As6526
YN2018D
WIV16
Rs4874
Rs4081
Rs4237
Rs4247
SC2018
SARS-CoV
YNLF_31C
YNLF_34C
F46
LYRa11
Yunnan2011
HuB2013
279_2005
Rm1
Longquan_140
HKU3-1
HKU3-2
HKU3-5
HKU3-3
HKU3-4
HKU3-13
HKU3-6
HKU3-10
HKU3-9
HKU3-11
HKU3-7
HKU3-8
HKU3-12
RpShaanxi2011
JL2012
JTMC15
HeB2013
SX2013
Rf1
Jiyuan-84

RBP region 7

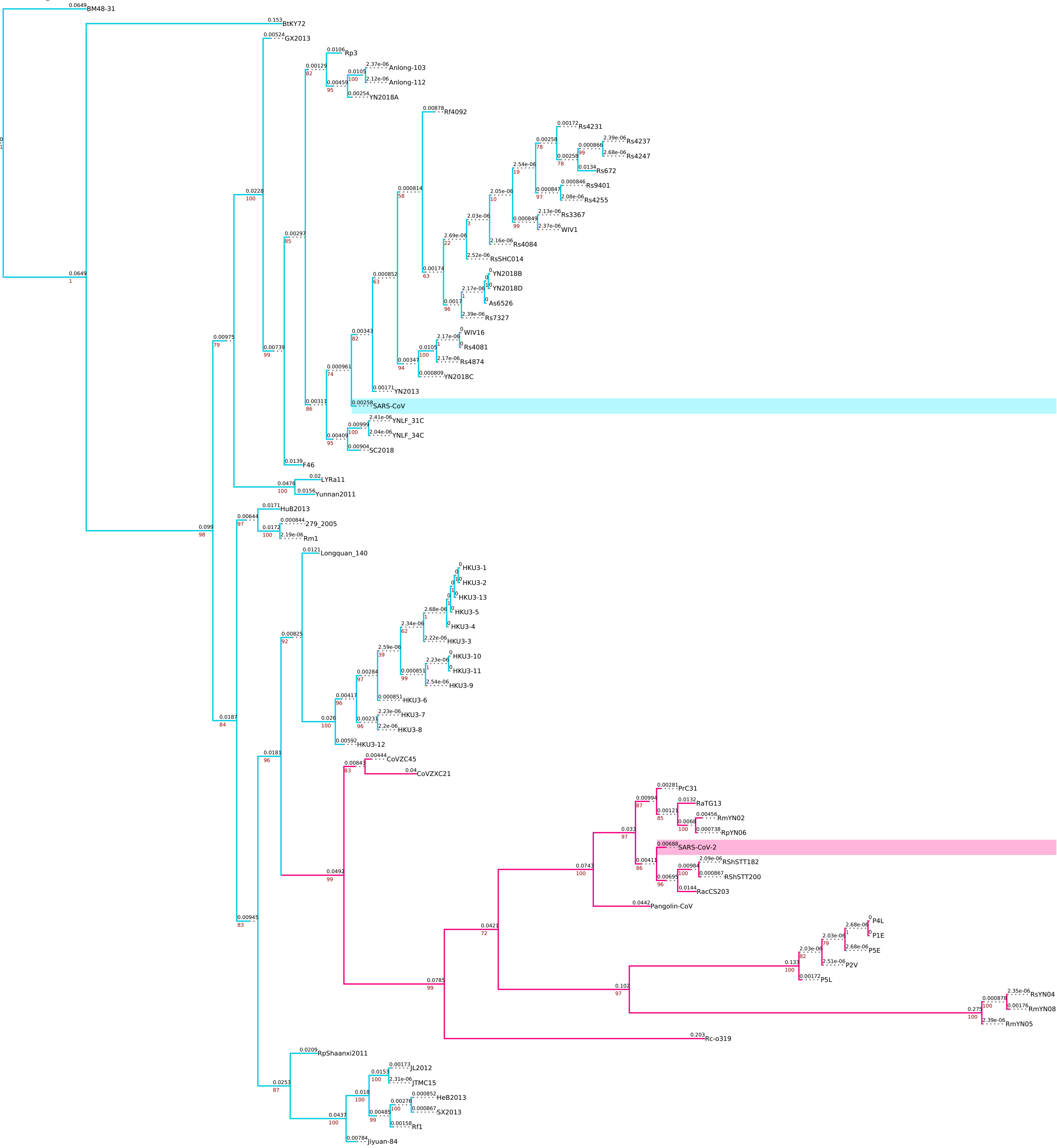

BM48-31
BtKY72
GX2013
Rp3
Anlong-103
Anlong-112
YN2018A
Rf4092
Rs4231
Rs4237
Rs4247
Rs672
Rs9401
Rs4255
Rs3367
WIV1
Rs4084
RsSHC014
YN2018B
YN2018D
As6526
Rs7327
WIV16
Rs4081
Rs4874
YN2018C
YN2013
SARS-CoV
YNLF_31C
YNLF_34C
SC2018
F46
LYRa11
Yunnan2011
HuB2013
279_2005
Rm1
Longquan_140
HKU3-1
HKU3-2
HKU3-13
HKU3-5
HKU3-4
HKU3-3
HKU3-10
HKU3-11
HKU3-9
HKU3-6
HKU3-7
HKU3-8
HKU3-12
CoVZC45
CoVZXC21
PrC31
RaTG13
RmYN02
RpYN06
SARS-CoV-2
RShSTT182
RShSTT200
RacCS203
Pangolin-CoV
P4L
P1E
P5E
P2V
P5L
RsYN04
RmYN08
RmYN05
Rc-o319
RpShaanxi2011
JL2012
JTMC15
HeB2013
SX2013
Rf1
Jiyuan-84

RBP region 8

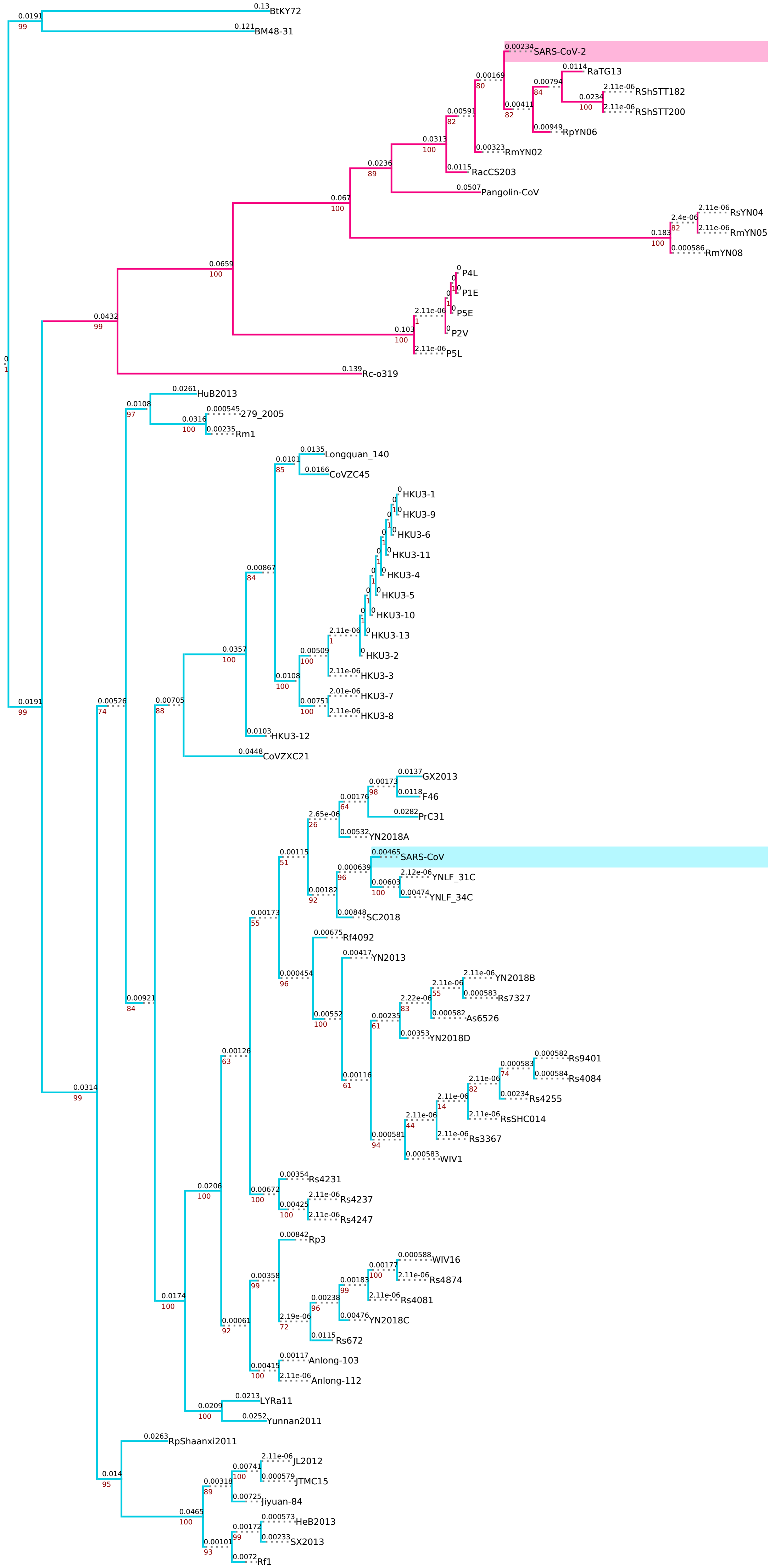
BtKY72
BM48-31
SARS-CoV-2
RaTG13
RShSTT182
RShSTT200
RpYN06
RmYN02
RacCS203
Pangolin-CoV
RsYN04
RmYN05
RmYN08
P4L
P1E
P5E
P2V
P5L
Rc-o319
HuB2013
279_2005
Rm1
Longquan_140
CoVZC45
HKU3-1
HKU3-9
HKU3-6
HKU3-11
HKU3-4
HKU3-5
HKU3-10
HKU3-13
HKU3-2
HKU3-3
HKU3-7
HKU3-8
HKU3-12
CoVZXC21
GX2013
F46
PrC31
YN2018A
SARS-CoV
YNLF_31C
YNLF_34C
SC2018
Rf4092
YN2013
YN2018B
Rs7327
As6526
YN2018D
Rs9401
Rs4084
Rs4255
RsSHC014
Rs3367
WIV1
Rs4231
Rs4237
Rs4247
Rp3
WIV16
Rs4874
Rs4081
YN2018C
Rs672
Anlong-103
Anlong-112
LYRa11
Yunnan2011
RpShaanxi2011
JL2012
JTMC15
Jiyuan-84
HeB2013
SX2013
Rf1

RBP region 9

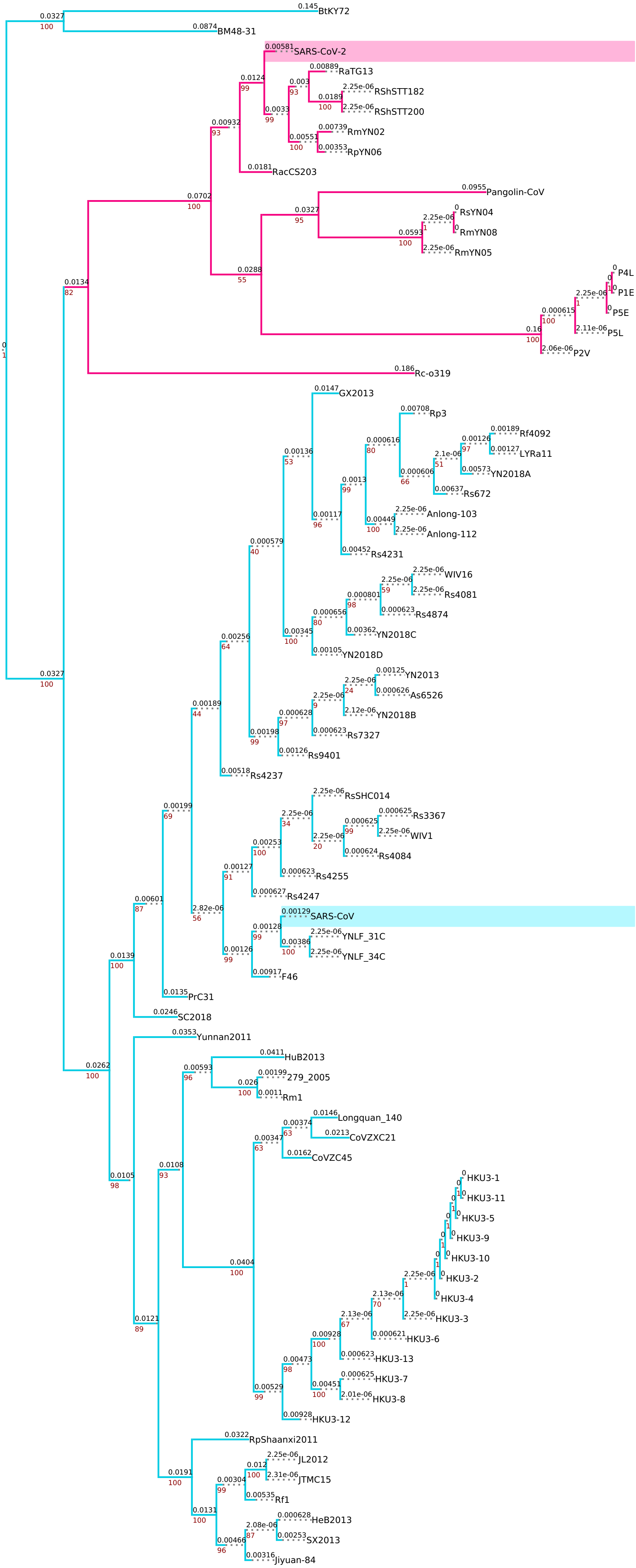
BtKY72
BM48-31
SARS-CoV-2
RaTG13
RShSTT182
RShSTT200
RmYN02
RpYN06
RacCS203
Pangolin-CoV
RsYN04
RmYN08
RmYN05
P4L
P1E
P5E
P5L
P2V
Rc-o319
GX2013
Rp3
Rf4092
LYRa11
YN2018A
Rs672
Anlong-103
Anlong-112
Rs4231
WIV16
Rs4081
Rs4874
YN2018C
YN2018D
YN2013
As6526
YN2018B
Rs7327
Rs9401
Rs4237
RsSHC014
Rs3367
WIV1
Rs4084
Rs4255
Rs4247
SARS-CoV
YNLF_31C
YNLF_34C
F46
PrC31
SC2018
Yunnan2011
HuB2013
279_2005
Rm1
Longquan_140
CoVZXC21
CoVZC45
HKU3-1
HKU3-11
HKU3-5
HKU3-9
HKU3-10
HKU3-2
HKU3-4
HKU3-3
HKU3-6
HKU3-13
HKU3-7
HKU3-8
HKU3-12
RpShaanxi2011
JL2012
JTMC15
Rf1
HeB2013
SX2013
Jiyuan-84

RBP region 10

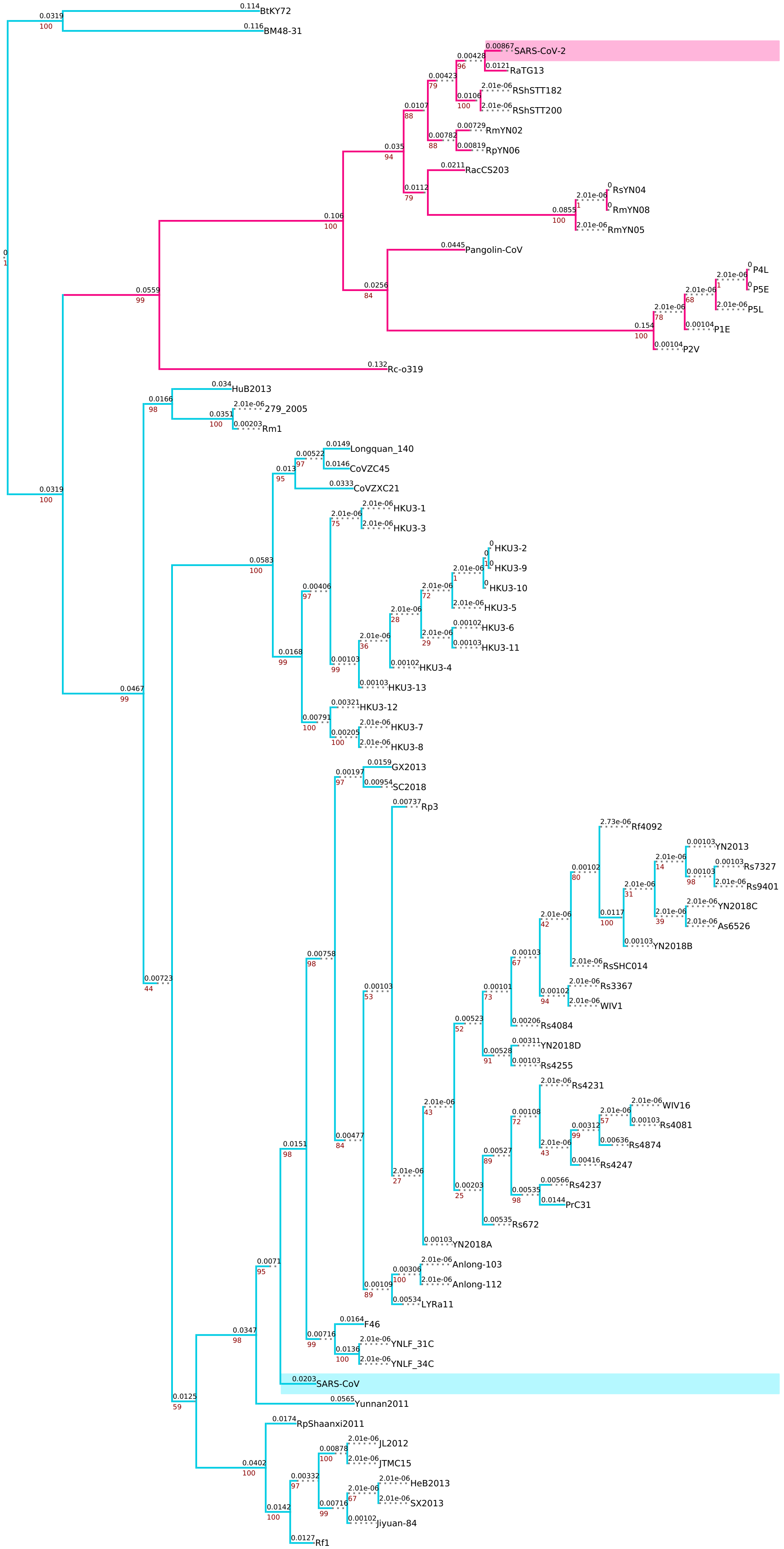

RBP region 11

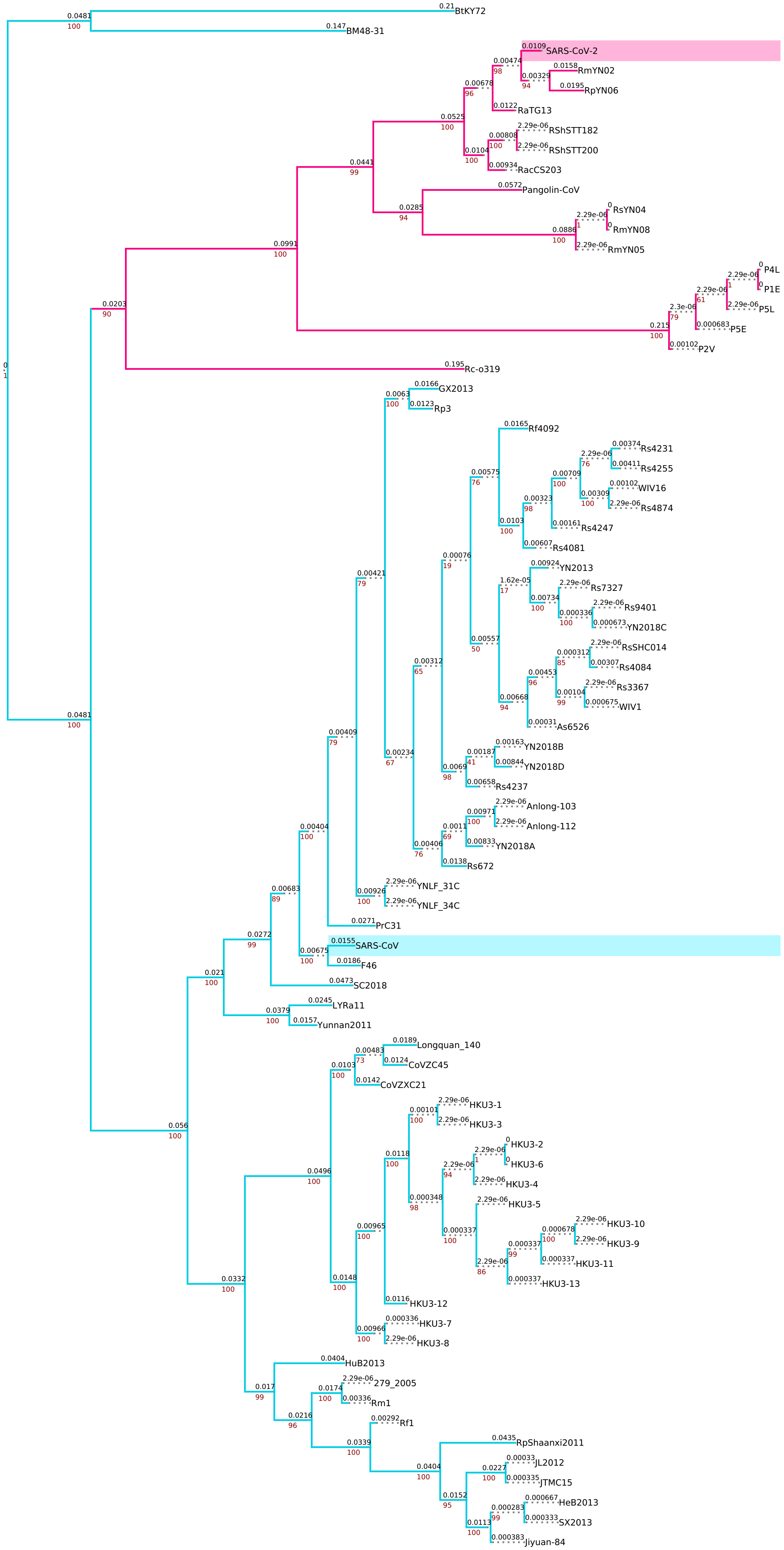

RBP region 12

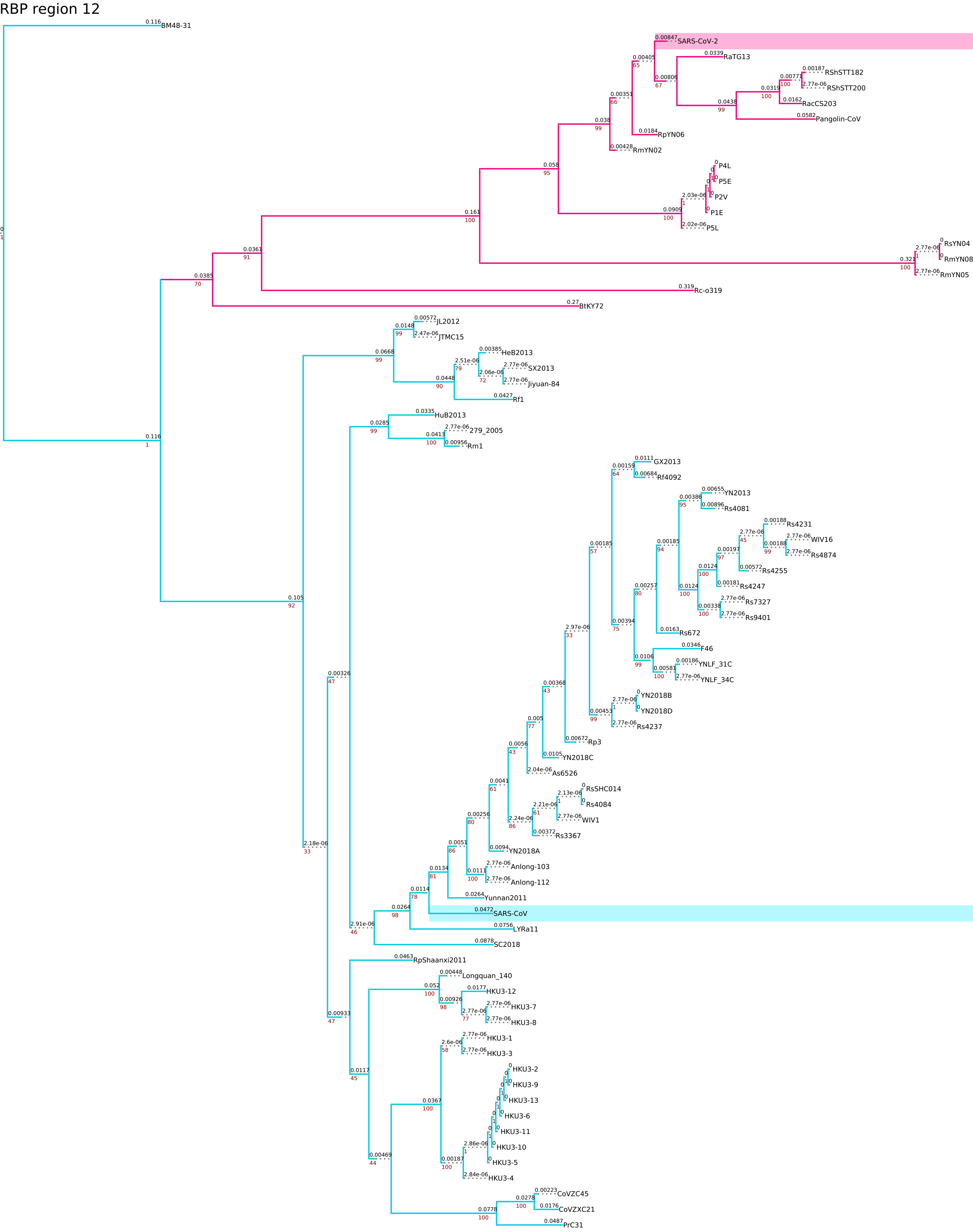

RBP region 13

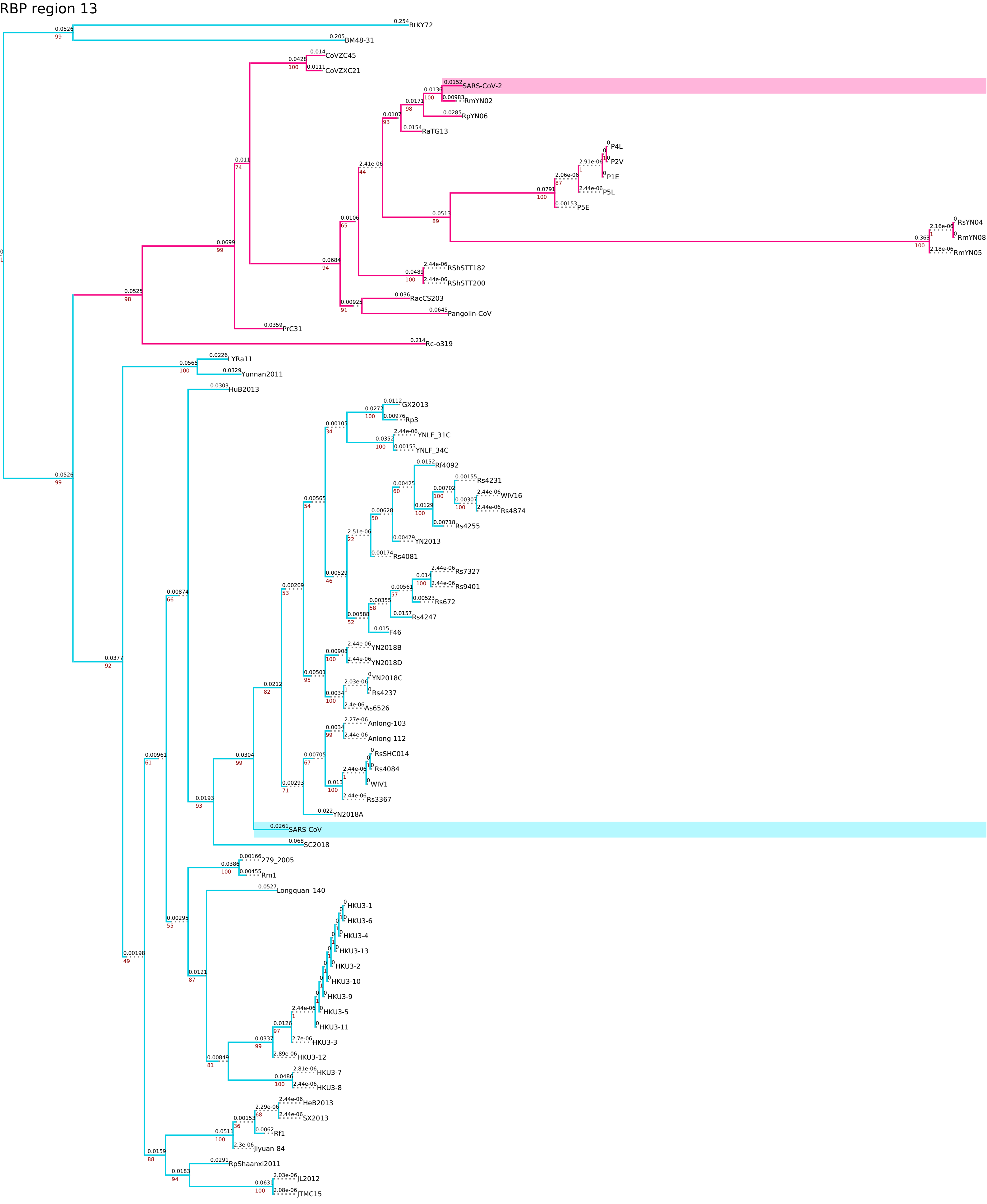

RBP region 14

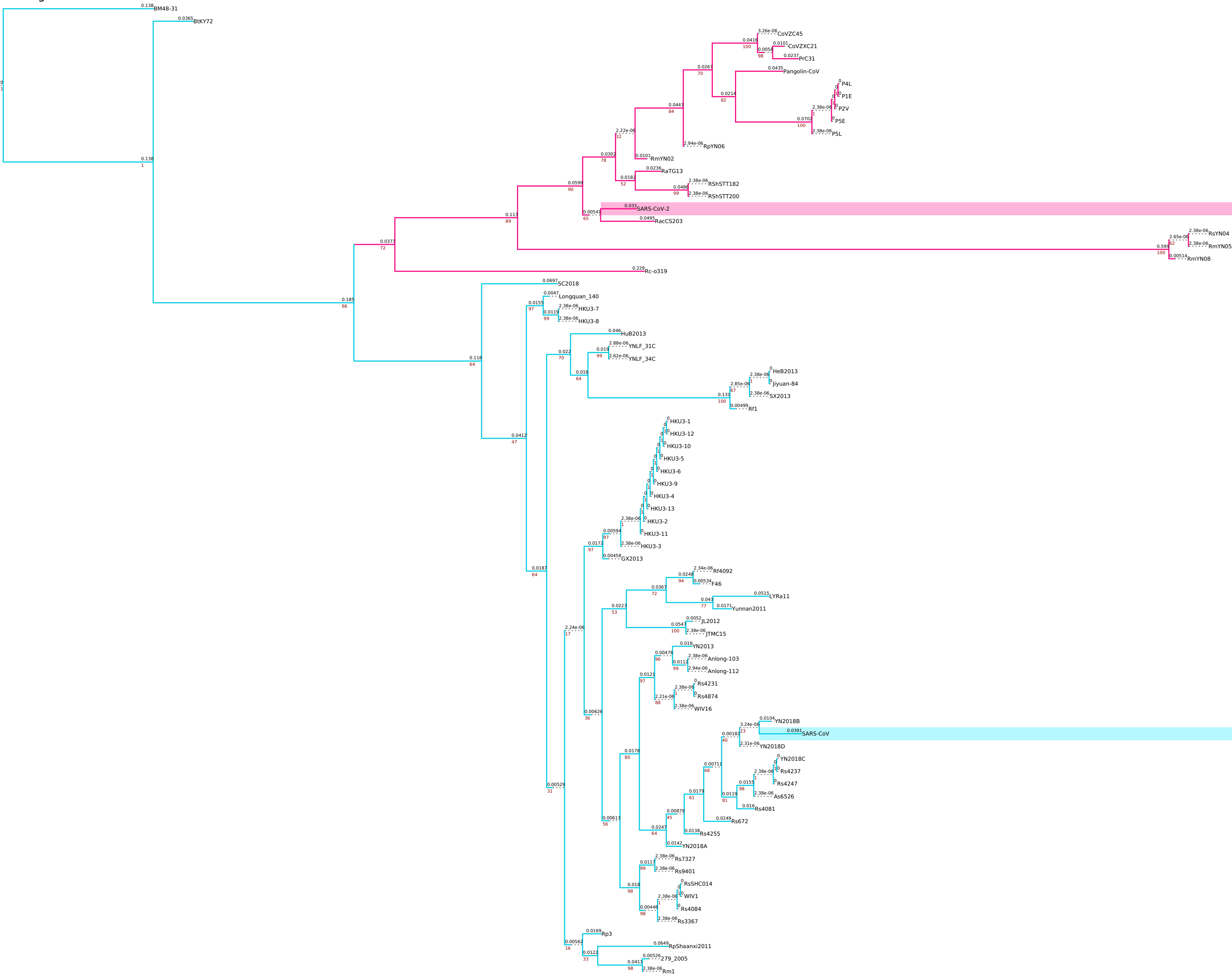

RBP region 15

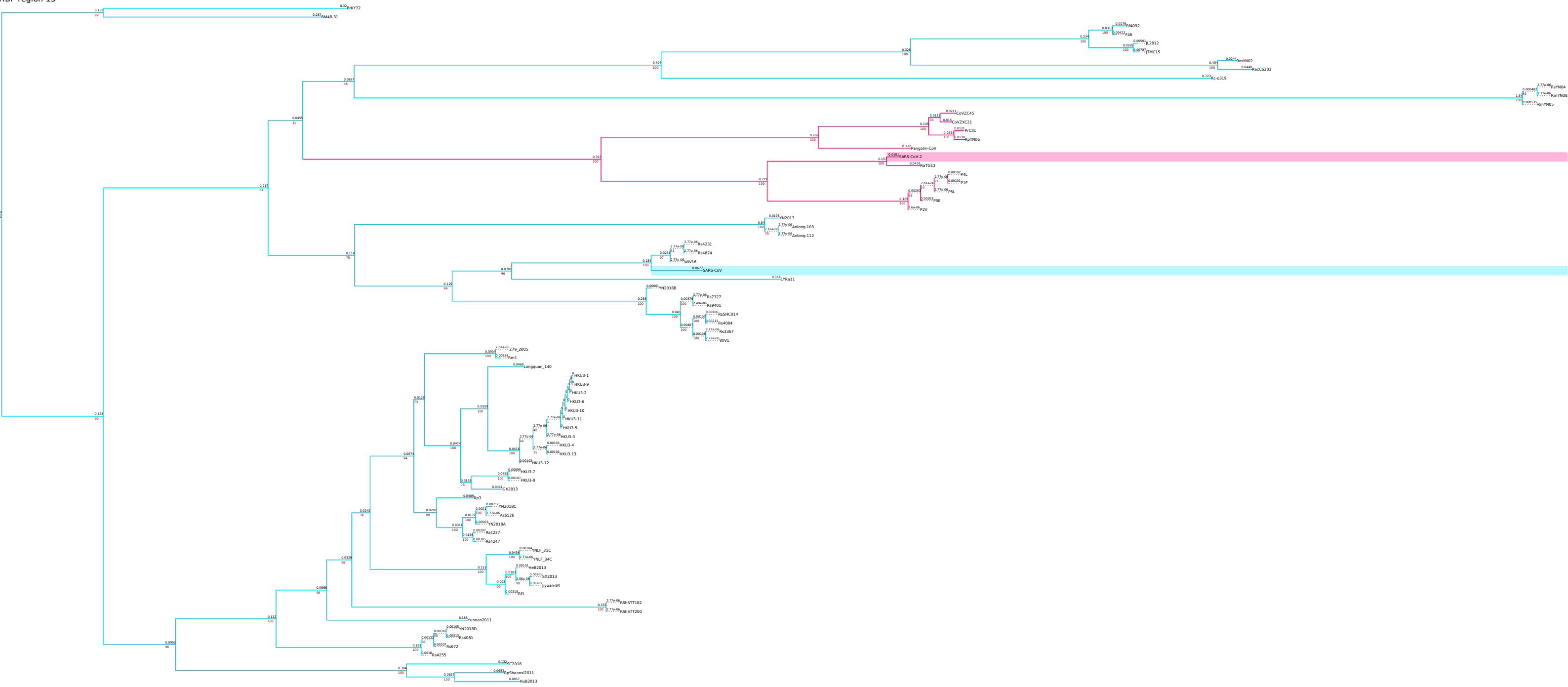

RBP region 16

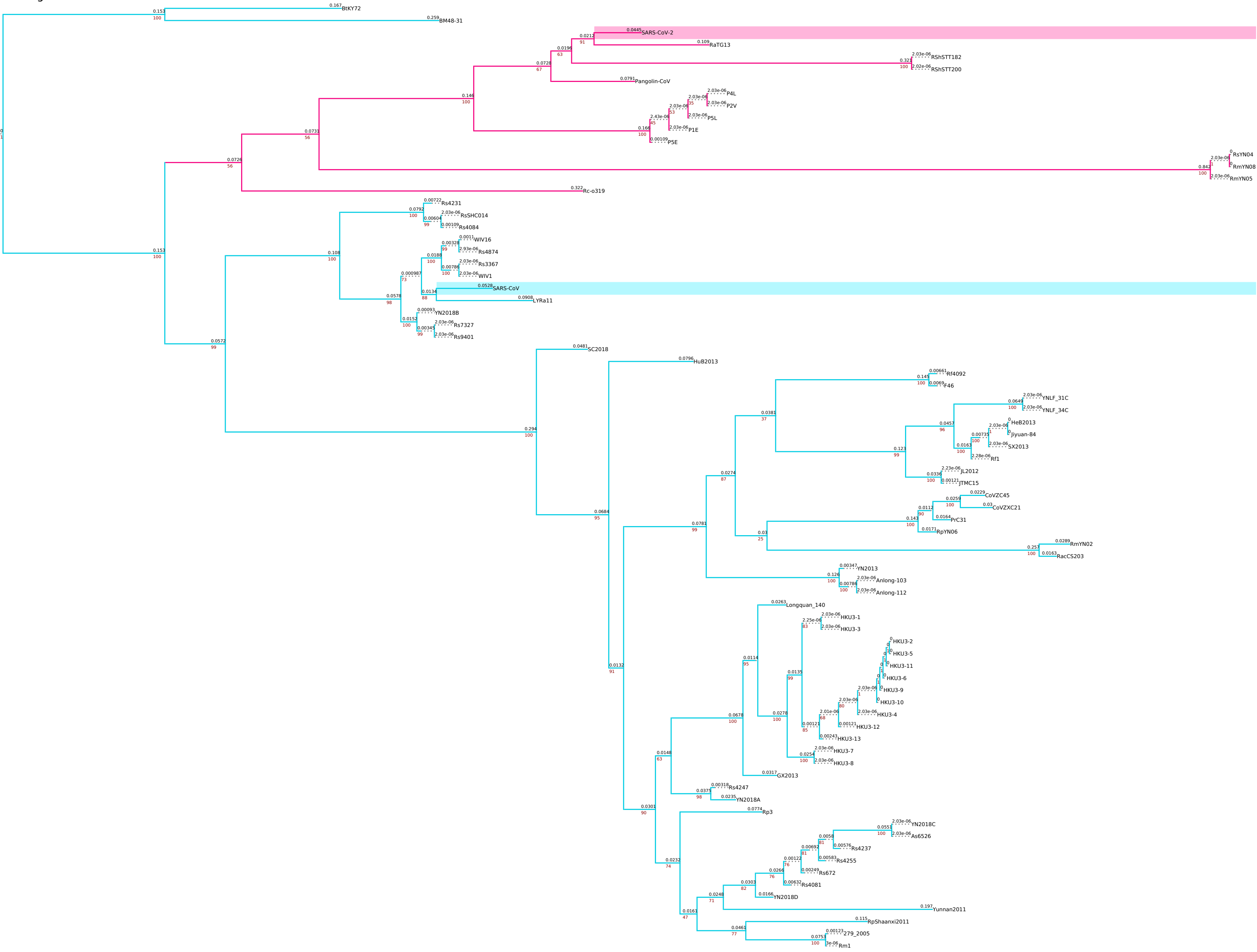

RBP region 17

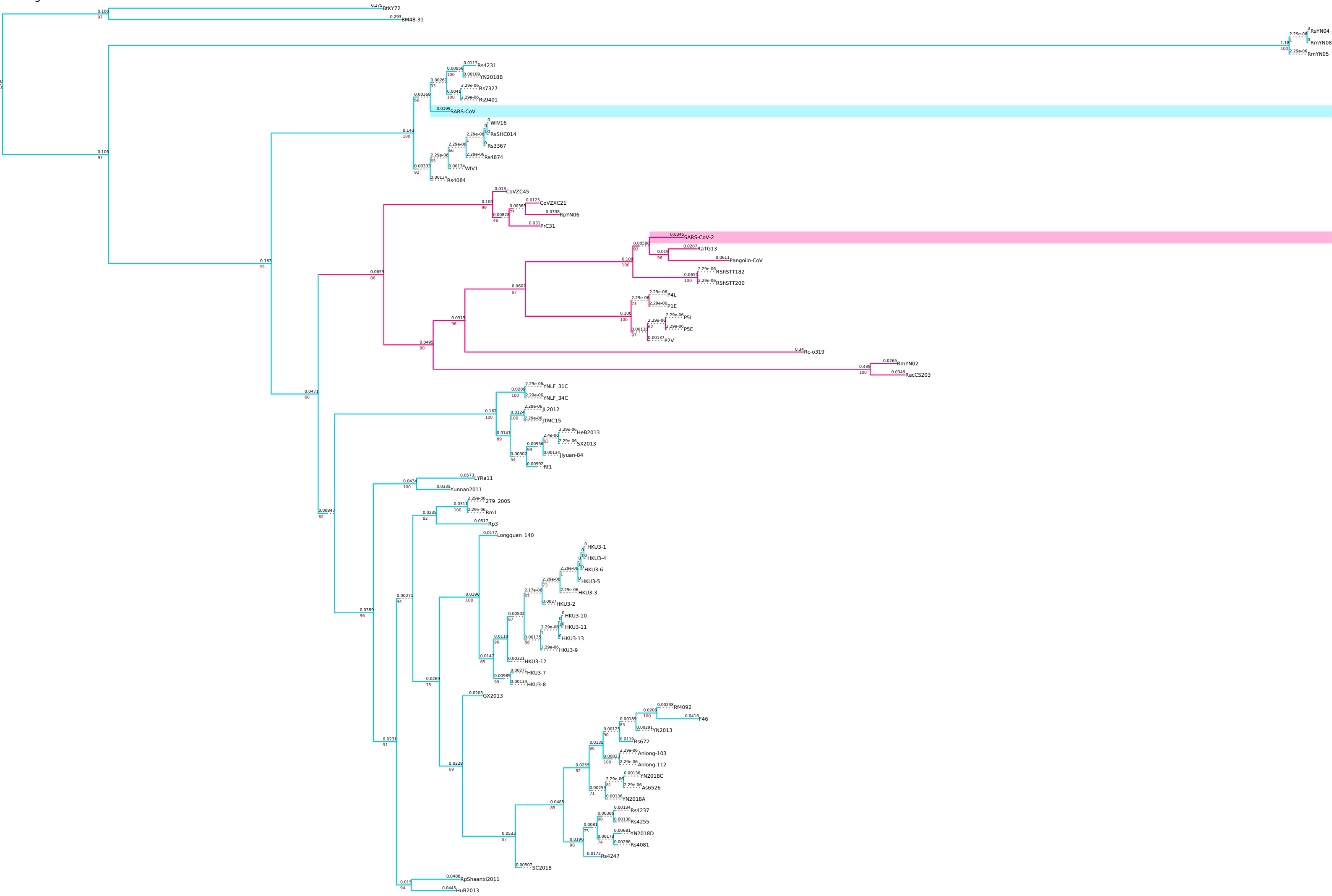

RBP region 18

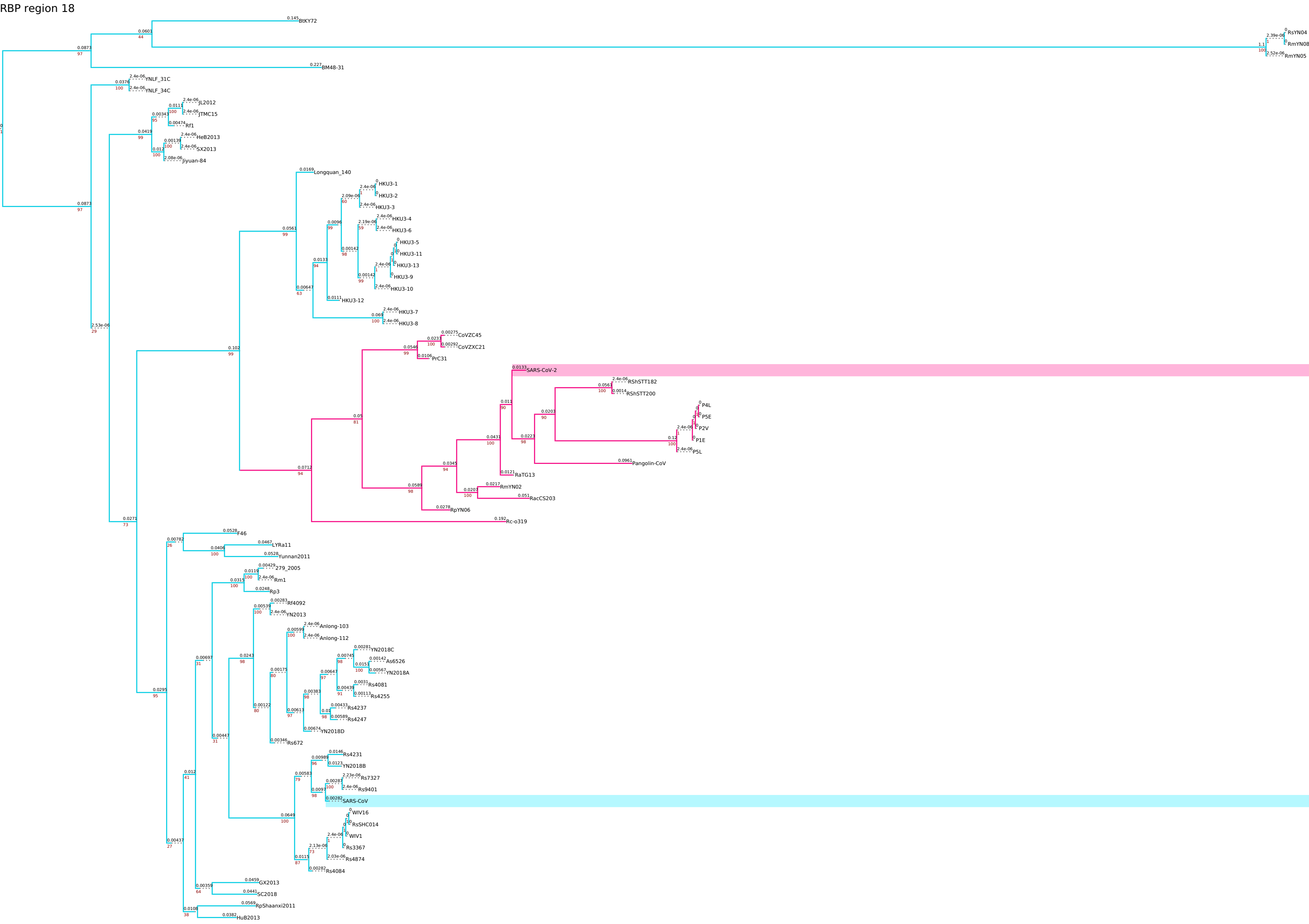

RBP region 19

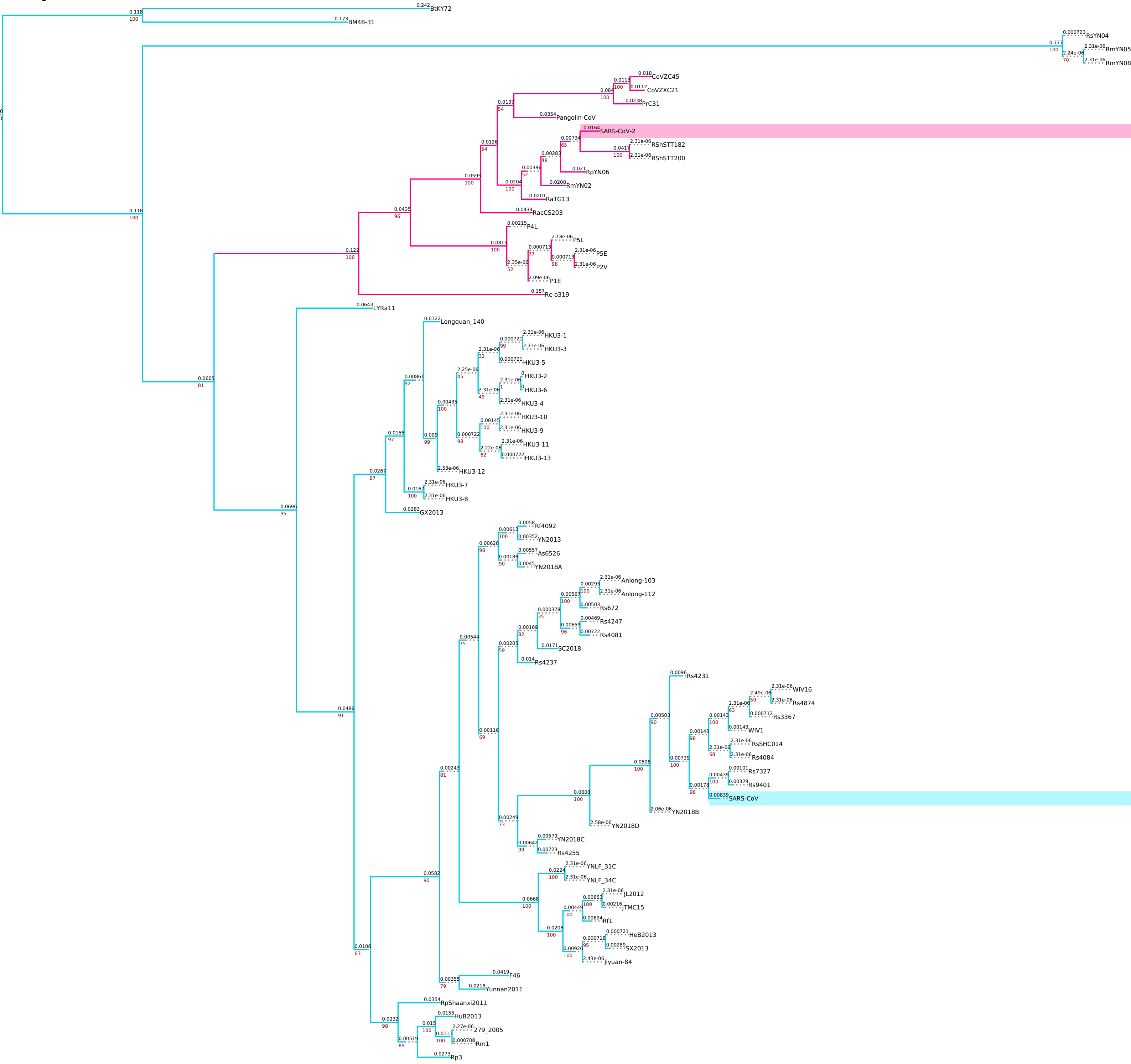

RBP region 20

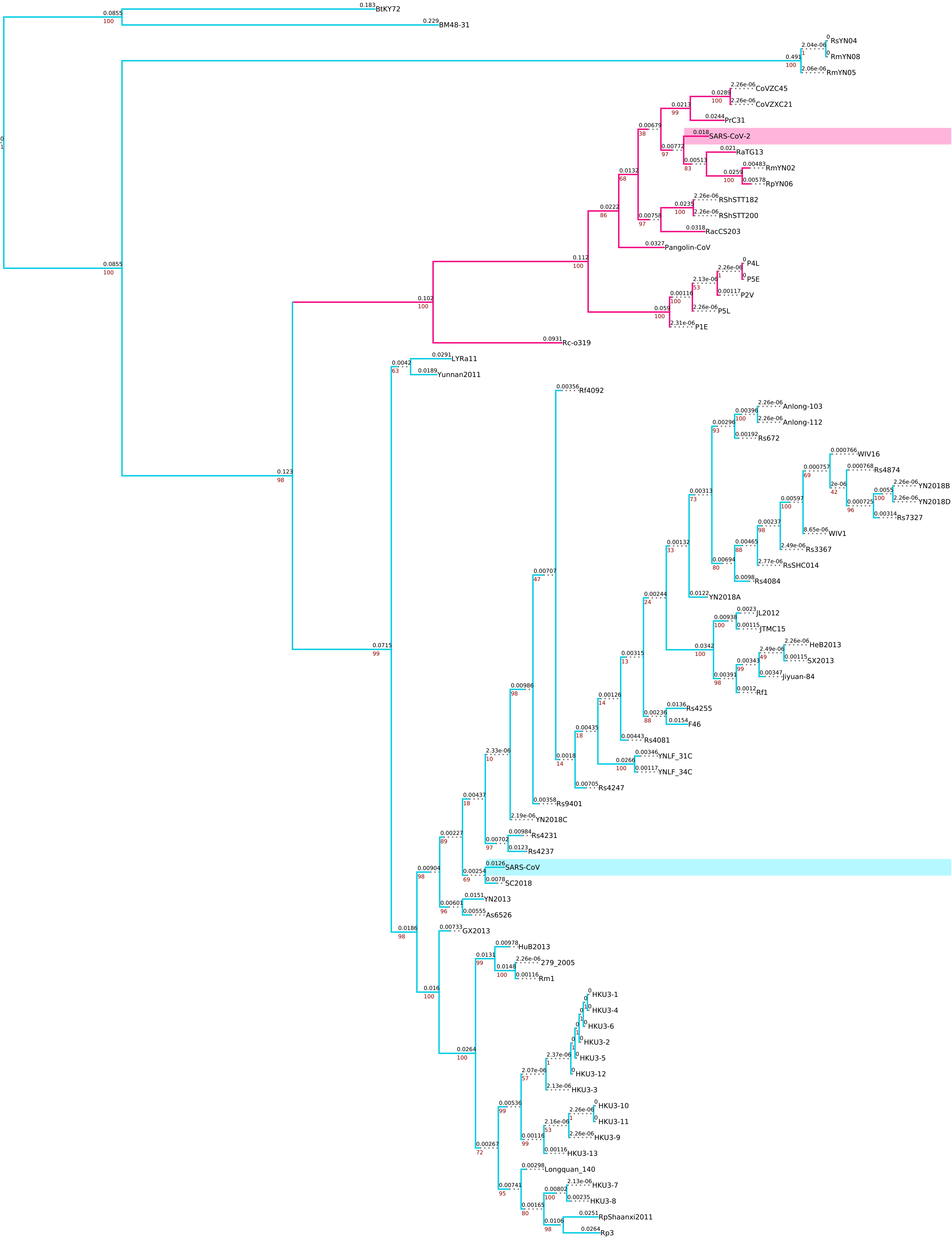

RBP region 21

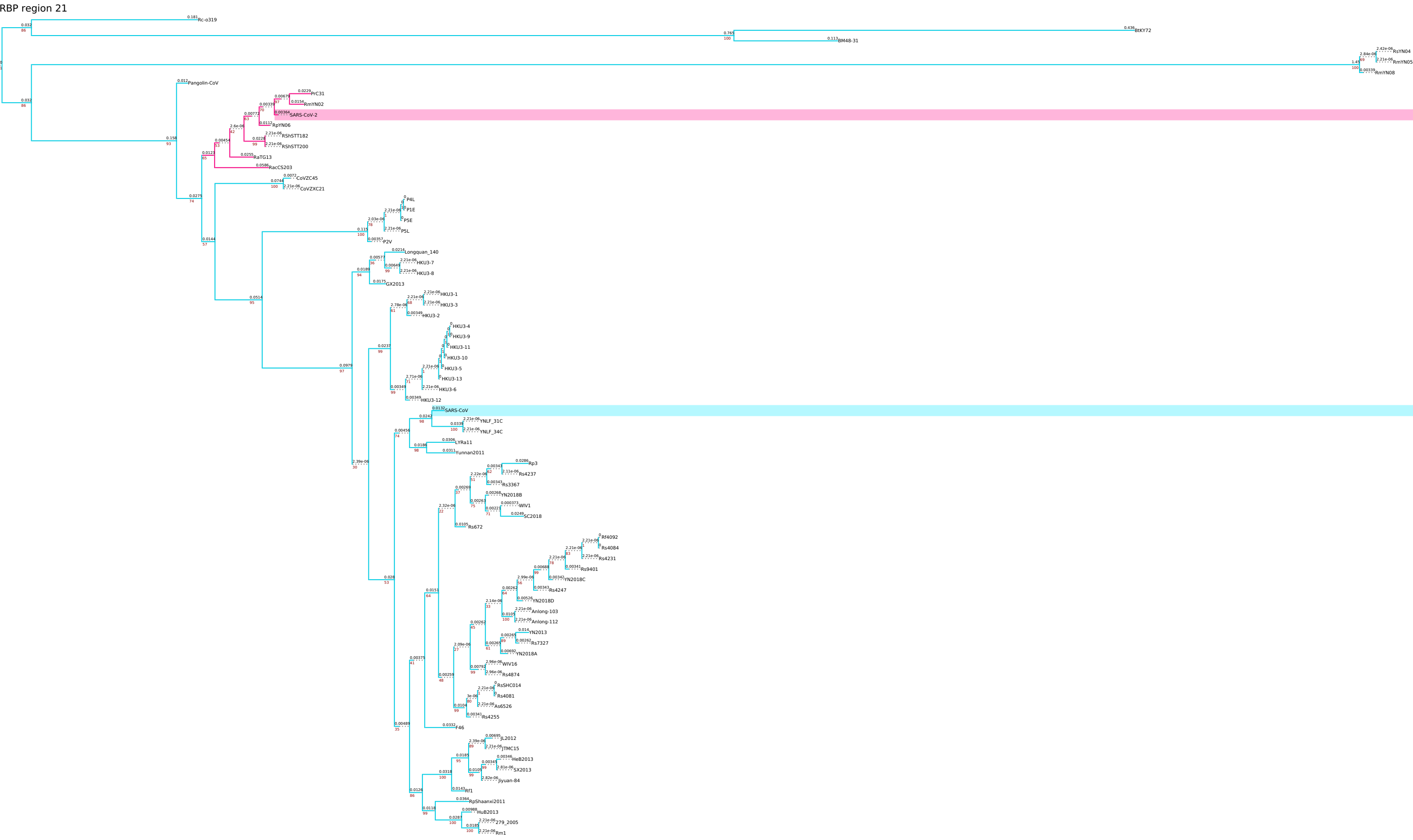

RBP region 22

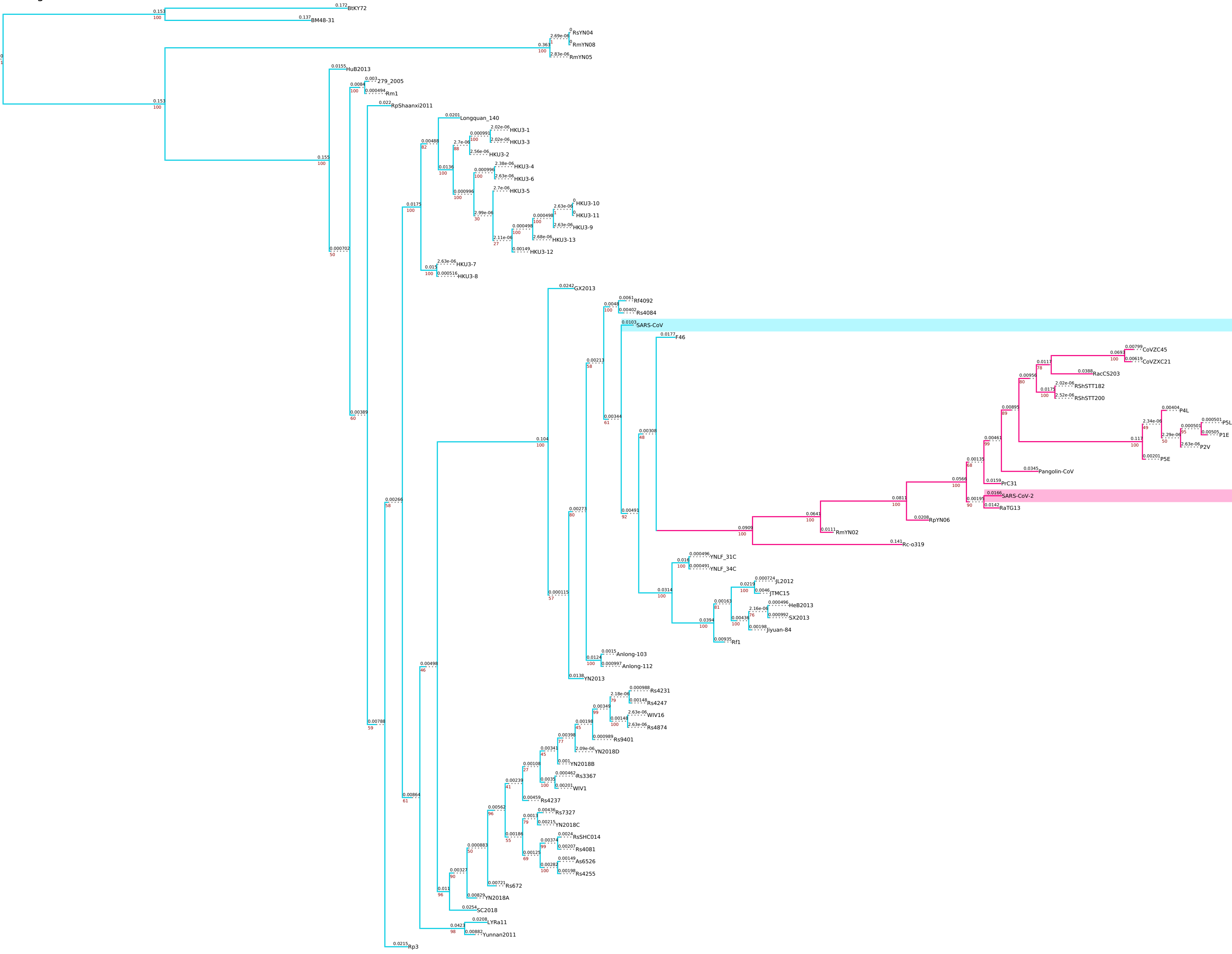

2 Figure S2

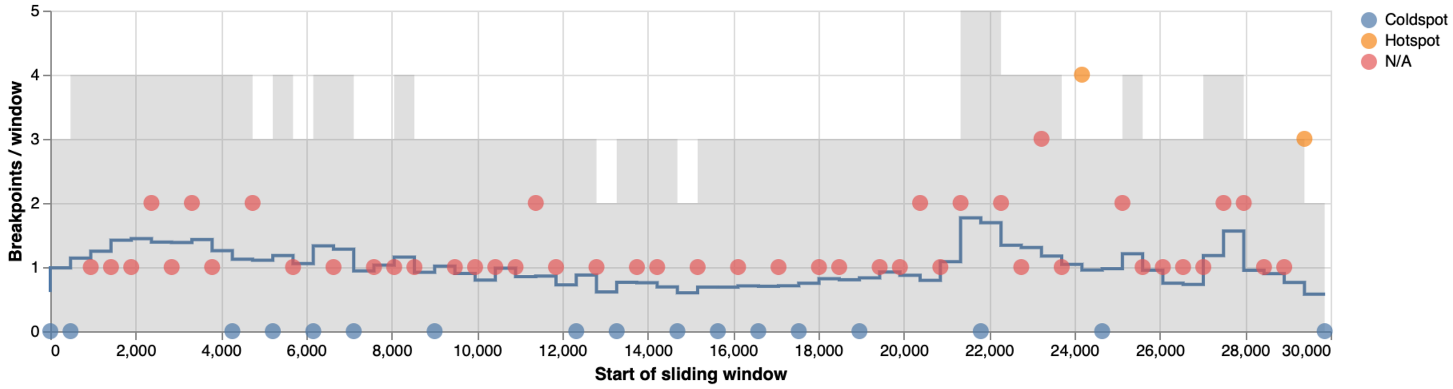

Figure S2. Permutation test for assessing potential clustering of the recombination breakpoints inferred by GARD. The blue line represents the mean of the number of breakpoints in the window (proportional to the density of variable sites). Grey shading shows the 2.5% - 97.5% intervals of breakpoints in each window. The number of inferred breakpoints in each window is shown in dots, in red if they fall within the permutation intervals (N/A), blue if they represent recombination coldspots (in the left tail of the permutation distribution) and orange if they represent recombination hotspots (in the right tail of the distribution).

3 Figure S3

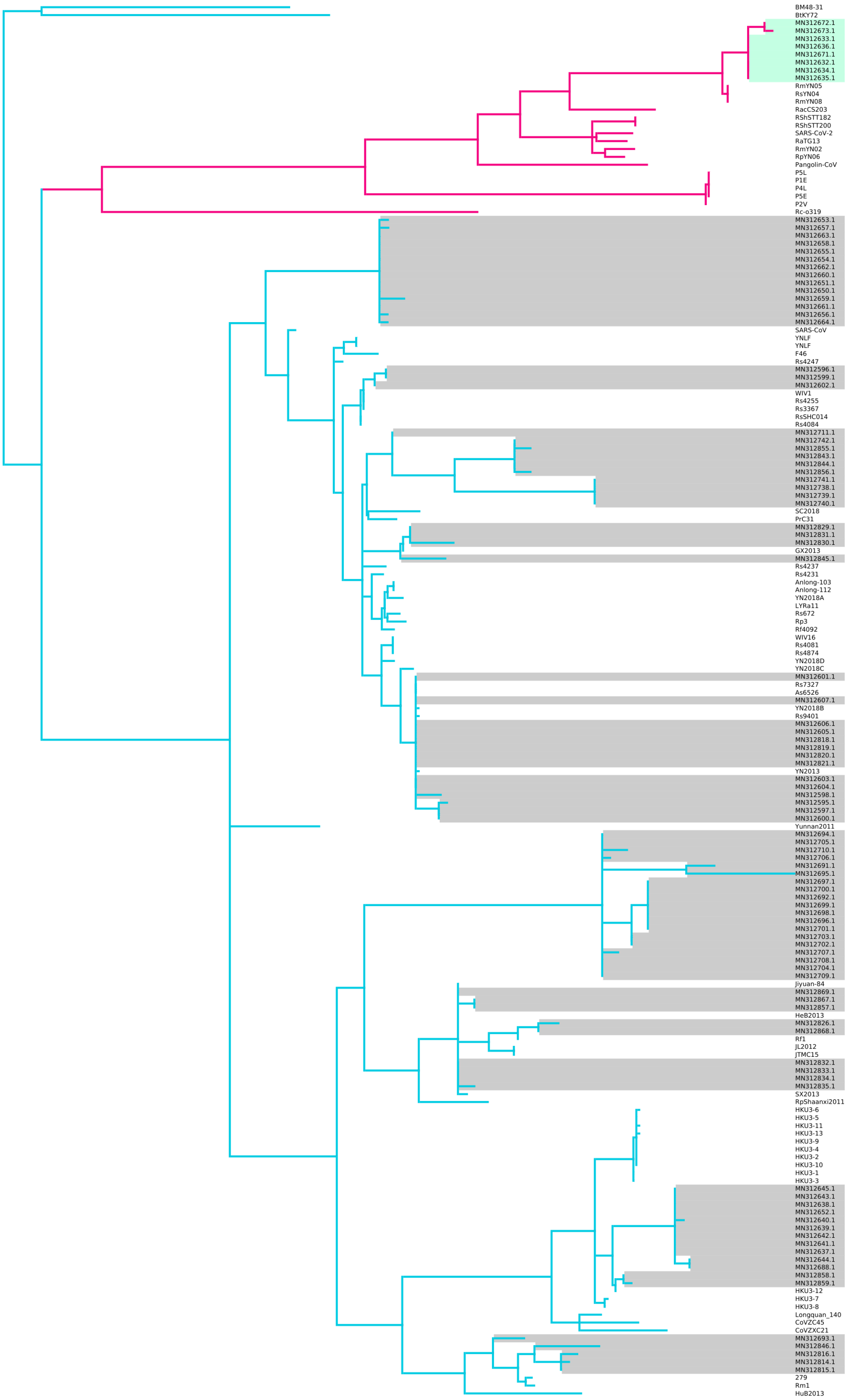

Figure S3. Maximum likelihood phylogeny reconstructed using iqtree (GTR+I+Γ4) of all 78 *Sarbecoviruses* used throughout the analysis, including the short RdRp fragments of related *Sarbecoviruses* reported in Latinne *et al.* (2020). The genomic region used for the alignment corresponds to the SARS-CoV-2 reference genome's Wuhan-Hu-1 coordinates 15280 - 16282. Nodes with bootstrap support (10,000 replicates) below 80 have been collapsed. The nCoV clade is annotated in pink and the non-nCoV clade in blue. SARS-CoV-2 and SARS-CoV are highlighted in pink and blue respectively. Viruses from Latinne *et al.* are highlighted in grey, apart from the 7 sequences that cluster within the nCoV clade which are highlighted in green. Out of this cluster of sequences MN312634.1 has been collected from a confirmed *R. affinis* bat species.
